# Zetomipzomib (KZR-616) attenuates lupus in mice via modulation of innate and adaptive immune responses

**DOI:** 10.3389/fimmu.2023.1043680

**Published:** 2023-03-10

**Authors:** Tony Muchamuel, R. Andrea Fan, Janet L. Anderl, Darrin J. Bomba, Henry W. B. Johnson, Eric Lowe, Brian B. Tuch, Dustin L. McMinn, Beatriz Millare, Christopher J. Kirk

**Affiliations:** Department of Research, Kezar Life Sciences, South San Francisco, CA, United States

**Keywords:** immunoproteasome, plasma cells, lupus nephritis (LN), SLE - systemic lupus erythematosus, autoimmunity

## Abstract

Zetomipzomib (KZR-616) is a selective inhibitor of the immunoproteasome currently undergoing clinical investigation in autoimmune disorders. Here, we characterized KZR-616 *in vitro* and *in vivo* using multiplexed cytokine analysis, lymphocyte activation and differentiation, and differential gene expression analysis. KZR-616 blocked production of >30 pro-inflammatory cytokines in human peripheral blood mononuclear cells (PBMCs), polarization of T helper (Th) cells, and formation of plasmablasts. In the NZB/W F1 mouse model of lupus nephritis (LN), KZR-616 treatment resulted in complete resolution of proteinuria that was maintained at least 8 weeks after the cessation of dosing and was mediated in part by alterations in T and B cell activation, including reduced numbers of short and long-lived plasma cells. Gene expression analysis of human PBMCs and tissues from diseased mice revealed a consistent and broad response focused on inhibition of T, B, and plasma cell function and the Type I interferon pathway and promotion of hematopoietic cell lineages and tissue remodeling. In healthy volunteers, KZR-616 administration resulted in selective inhibition of the immunoproteasome and blockade of cytokine production following *ex vivo *stimulation. These data support the ongoing development of KZR-616 in autoimmune disorders such as systemic lupus erythematosus (SLE)/LN.

## 1 Introduction

SLE is a systemic autoimmune disorder that severely affects multiple organs including the kidney, where organ damage resulting from LN is a major risk factor for mortality (1, 2). SLE pathogenesis involves a complex interplay of genetic, immunologic, and environmental factors leading to dysregulated innate and adaptive immunity resulting in heterogeneous clinical manifestations and a waxing and waning course of disease. The molecular sequelae underlying SLE and LN remain to be fully elucidated, but include defective clearance of apoptotic cellular components, a breakdown in induction of T cell tolerance, generation of antibodies against intracellular components [e.g., anti-double-stranded DNA (anti-dsDNA)], and aberrant expression of multiple inflammatory cytokines. The heterogeneity of immune dysregulation in SLE patients has been demonstrated by gene expression profiling studies (3, 4).

Development of novel therapeutic agents for SLE has been extensive, but despite decades of effort, results have been relatively disappointing, indicating the urgent need to identify and validate new therapeutic targets (5). In particular, agents targeted to individual cytokines or cell types have resulted in only incremental improvements in patient outcomes relative to current standards of care, which involve the use of corticosteroids and broad immunosuppressive agents such as the anti-mitotic agent mycophenolate mofetil (MMF). Novel agents targeting multiple immune pathways may improve efficacy in a broader population of patients with SLE (6).

Selective inhibitors of the immunoproteasome have emerged as an attractive therapeutic target in the treatment of immune-mediated disorders, including autoimmune diseases (7–10). The immunoproteasome is a distinct class of 26S proteasome and is characterized by the expression of 3 proteolytic active sites found in the 20S core particle: low molecular mass polypeptide (LMP)7, LMP2, and multicatalytic endopeptidase complex-like 1 (MECL-1). Basal expression of the immunoproteasome is primarily restricted to cells of hematopoietic origin, but is induced at sites of inflammation, including the kidneys of patients with LN (11). Selective inhibitors of active site subunits, such as ONX 0914 (formerly known as PR-957), have been described and used to probe the role of the immunoproteasome in immune responses. In mouse models, ONX 0914 reduced the number and activity of inflammatory T cell subsets (such as Th1 and Th17), increased the number of regulatory T cells (Treg), blocked autoantibody formation, and attenuated disease progression in models of autoimmune diseases (including rheumatoid arthritis, inflammatory bowel disease, multiple sclerosis, and SLE) (12–15). More recently, multiple groups have shown that inhibition of both LMP7 and LMP2 are required to replicate the anti-inflammatory activity of ONX 0914 (16, 17). From these findings, we generated KZR-616, a tripeptide ketoepoxide and an analog of ONX 0914 that selectively and potently inhibits both LMP7 and LMP2 *in vitro* and *in vivo* and is therapeutically active in a mouse model of rheumatoid arthritis (**Supplemental Figure 1**) (17). KZR-616 demonstrated higher potency for LMP2 relative to ONX 0914 and a >10,000-fold increase in solubility, thus enabling subcutaneous administration (17).

Here we report the effects of KZR-616 on human leukocytes *in vitro* and in mouse models of SLE, demonstrating the therapeutic potential of this agent and the molecular mechanisms of its anti-inflammatory activity. KZR-616 treatment in NZB/W F1 diseased mice resulted in a complete and durable resolution of proteinuria and significant reductions in autoantibody production and renal IgG deposition. We found that KZR-616-driven resolution of nephritis in mice was associated with changes in the expression of genes involved in multiple immune effector cell signaling pathways, plasma cell differentiation, mitochondrial and metabolic dysfunction, and glomerular injury. Many of these changes were also seen when human leukocytes from either healthy volunteers or patients with SLE were exposed to KZR-616. Furthermore, administration of KZR-616 to healthy volunteers resulted in selective inhibition of the immunoproteasome and impaired cytokine responses to mitogenic stimulation under *ex vivo* stimulation conditions. Taken together, our data suggest that KZR-616 has the potential to be therapeutically active in LN and other related autoimmune indications through a broad immunomodulatory response.

## 2 Materials and methods

### 2.1 Cells and compounds

Purified PBMCs, naïve CD4^+^ T cells, and CD19^+^ B cells (isolated by positive selection) from healthy donors were purchased from AllCells. Matched (age, gender, race, **Table S1**) SLE patient and healthy donor PBMCs were acquired from Conversant (Now Discovery Life Sciences). KZR-616 stock was dissolved in DMSO; DMSO was used as vehicle control for *in vitro* and *ex vivo* studies.

### 2.2 PBMC stimulation and cytokine/RNA analysis

PBMCs (200,000 cells/well) were plated in culture media in 96-well round bottom plates. After a 1 hour incubation with KZR-616 at 37°C, 5% CO_2_, plates were washed 4 times with culture media and cells were stimulated with either LPS (Escherichia coli, O111:B4, 1 µg/mL, MilliporeSigma), plate-bound antibodies to CD3 (clone OKT3, 5 µg/mL, Thermo Fisher Scientific) and soluble CD28 (µg/mL, clone CD28.2, BD Biosciences), or phytohemagglutinin (PHA, 10 µg/mL, MilliporeSigma) for 24 hours, or phorbol 12-myristate 13-acetate (PMA, 50 ng/mL, MilliporeSigma) and ionomycin (1 µg/mL, MilliporeSigma) for 6 hours. Supernatants were collected and analyzed for cytokine release by multiplexed electrochemiluminescent immunoassay detection (44-plex cytokine/chemokine panel, Meso Scale Diagnostics). PBMC pellets were lysed in RLT buffer with 2-mercaptoethanol (Qiagen) followed by total RNA extraction for RNA-Seq (Q2 EA solutions).

### 2.3 T helper differentiation and analysis

Naïve CD4^+^ T cells were treated with KZR-616 as described above for PBMCs and cultured for up to 6 days at 37°C with 5% CO_2_ in RPMI 1640 media, supplemented with 10% FBS (both from Mediatech) along with antibodies to CD3 and CD28 (same as above) in Th0 (no cytokines), Th1 [human IL-2 (2 ng/ml), IL-12 (50 ng/ml), anti-IL-4 (5 µg/ml)], Th2 [human IL-2 (2 ng/ml), IL-4 (50 ng/ml), anti-IFN-γ (5 µg/ml)], Th17 [IL-23 (30 ng/ml), IL-6 (50 ng/ml), IL-1β (20 ng/ml), TGF-β (10 ng/ml), anti-IFN-γ (5 µg/ml), anti-IL-4 (5 µg/ml)], or Treg [human IL-2 (5 ng/ml), TGF-β (10 ng/ml)] polarizing conditions. Cytokines and antibodies were purchased from BD Biosciences or R&D Systems (Minneapolis, MN). Cells were analyzed by flow cytometry for intracellular cytokines and FoxP3 following the manufactures’ protocol using standard kits (BD Biosciences). Flow cytometry was performed on BD FACSVerse™ System with BD FACSuite™ Software for acquisition, and data analysis was done using BD FlowJo™ Software. Supernatants were collected and analyzed for cytokines (IFN-γ, IL-4, and IL-17) by multiplex MSD immunoassay following the manufacture’s protocol (Meso Scale Diagnostics).

### 2.4 Human plasmablast differentiation and analysis

Human peripheral B cells were treated with KZR-616 as described above for PBMCs and stimulated for 6 days at 0.5x10^6^ cells/mL with 100 ng/mL IL-21 (R&D Systems), 1 µg/mL anti-CD40 (R&D systems), and 5 ug/mL anti-IgM (Jackson ImmunoResearch) to induce plasmablast differentiation. Cells were analyzed by flow cytometry for surface markers including CD19, CD20, and CD38 (BD Biosciences). Supernatants were analyzed for IgG by MSD immunoassay (Meso Scale Diagnostics).

### 2.5 Mice and experimental design

NZB/W F1 and MRL/lpr mice were supplied by Jackson Laboratories (Bar Harbor, ME). Proteinuria was monitored monthly using Albustix (Bayer Corp). To analyze the therapeutic effect of KZR-616, male MRL/lpr (10 week) mice with established disease (proteinuria positive and anti-IgG dsDNA antibody positive) were treated with KZR-616 intravenously (IV) at 5 mg/kg or subcutaneously (SC) at 12 mg/kg three times per week or once a week at 7.5 mg/kg for 13 consecutive weeks. Similarly, female NZB/W F1 (24-26 weeks) mice with established disease (proteinuria positive, anti-IgG dsDNA antibody positive) were treated with vehicle (10% hydroxypropyl-beta cyclodextrin), KZR-616 IV at 5 mg/kg or SC at 10 mg/kg three times per week or once a week for 13 consecutive weeks. MMF was administered daily by oral gavage at 30 mg/kg either alone or in combination with KZR-616 administered SC at 5 mg/kg once a week.

### 2.6 Mouse serum ELISA assay

Total IgG levels were measured in serum samples using a commercially available ELISA kit (Abcam). Samples were also measured for antibodies to dsDNA *via* ELISA (Alpha Diagnostics International).

### 2.7 Mouse tissue flow cytometry analysis

For assessment of T and B cell populations in NZB/W F1 mice, splenocytes and/or bone marrow were harvested following drug treatment and analyzed by flow cytometry. Single immune cell suspensions were prepared from spleen or bone marrow after red blood cell lysis. Cells were incubated with anti-CD16/CD32 (Fc block, clone 2.4G2) and stained with various combinations of antibodies. Antibodies used for these analyses were murine-reactive CD21-FITC, B220-FITC, CD3-PerCPCy5.5, CD11b-PerCPCy5.5, Gr1-PerCPCy5.5, B220- PerCP, CD23-PECy7, B220-AlexFluor700, IgD-BV510, CD38- FITC, CD44-FITC, B220-FITC, CD69-PE, CD86-PE, CD138-PE, CD3- PerCPCy5.5, CD5-PerCPCy5.5, B220-PerCP, ICOS-PECy7, CD4-PB, B220-PB, B220-AlexFluor700, IgD-BV510, CD8-APC, Ki67-APC (all antibodies and Fc block from BD Biosciences). Flow cytometry was run on BD FACSVerse™ System with BD FACSuite™ Software for acquisition, and data analysis was done using BD FlowJo™ Software.

### 2.8 BrdU analysis

Short and long-lived plasma cells were analyzed as previously described (18, 19). Briefly, NZB/W F1 mice were fed with 1 mg/ml BrdU (Sigma) in the drinking water for 14 days before treatment with KZR-616 and continued BrdU treatment until analysis. After surface staining, we performed intracellular staining for κ light chain and incorporated BrdU with the BrdU-Flow-Kit (BD Biosciences) according to the manufacturer’s instructions.

### 2.9 Histological and immunohistochemical analysis

Kidneys from different treatment groups were fixed in 10% formalin, embedded in paraffin, sectioned and stained with H&E. Pathology was analyzed and scored in a blinded fashion; sections were evaluated for total renal injury (glomerulonephritis) as well as injury to individual renal components (glomeruli, tubules, interstitial and lymphoid infiltrates) in the NZB/W model and then for total renal injury (glomerulonephritis, infiltrates, glomerular lesions) in the MLR/lpr model. Severity of injury was graded utilizing a standard grading system whereby 0 = no significant change, 1 = minimal, 2 = mild, 3 = moderate and 4 = severe.

### 2.10 Immunohistochemical staining of kidney

To detect immunoglobulin IgG deposition in glomeruli, kidney sections were stained with a biotinylated goat anti-murine IgG (Jackson ImmunoResearch Labs). Sections were then washed and incubated with a streptavidin-linked horseradish peroxidase (Agilent). Bound antibodies were visualized following incubation with 3,3’-diaminobenzidine solution (0.05% with 0.015% H_2_O_2_ in PBS; Agilent). Sections were counterstained with Mayer’s hematoxylin.

### 2.11 Mouse tissue RNA extraction and preparation

RNA from frozen spleen and kidney were isolated by disrupting the tissues in RLT buffer using a tissue lyser and then extracting RNA using the RNeasy Mini kit (Qiagen), including the on-column DNase digestion. Total RNA concentration and RNA integrity of samples was determined using the NanoDrop 8000 (Thermo Scientific) and the Fragment Analyzer with High Sensitivity RNA kit (Advanced Analytical), respectively. The RNeasy Protect Animal Blood kit was used to purify total RNA from whole blood collected and stabilized in RNAProtect Animal Blood tubes per manufacturer’s instructions. Globin mRNA was depleted from total RNA samples using the GlobinClear-Mouse/Rat kit (Qiagen). The final globin mRNA-depleted RNA samples were quantitated by spectrophotometry prior to continuing to library preparation.

### 2.12 RNA sequencing

Sequencing libraries were created using the Illumina TruSeq Stranded mRNA method. Briefly, total RNA samples were concentration normalized, and poly-adenylated RNA was purified using oligo-dT attached to magnetic beads. Purified mRNA was fragmented using heat in the presence of divalent cations. The fragmented RNA was converted into double-stranded cDNA, with dUTP utilized in place of dTTP in the second strand master mix. The double stranded cDNA underwent end-repair, A-tailing, and ligation of adapters that included index sequences. The libraries were amplified *via* polymerase chain reaction (PCR) and purified. Final libraries were assessed using qPCR for quantitation and TapeStation for fragment size assessment. Normalized libraries were pooled and sequenced at a plex-level appropriate to the coverage required.

Libraries were sequenced using the Illumina sequencing-by-synthesis platform, with a sequencing protocol of 50 bp paired-end and total read depth of 30M reads per sample. Normalized libraries were multiplexed for efficient sequencing to the required number of reads per sample. Pooled libraries were bound to the surface of a flow cell, and each bound template molecule was clonally amplified up to 1000-fold to create individual clusters. During each sequencing cycle, four fluorescently labeled nucleotides flowed over the surface of the flow cell and incorporated into each nucleic acid chain. Each nucleotide label acted as a terminator for polymerization, thereby ensuring that a single base was added to each nascent chain during each cycle. Fluorescence was measured for each cluster during each cycle to identify the base that was added to each cluster. The dye was then enzymatically removed to allow incorporation of the next nucleotide during the next cycle.

### 2.13 Alignment and quantification

Alignment and quantification were performed with STAR version 2.4 and RSEM version 1.2.14 as described previously (20, 21).

### 2.14 Differential expression and module/pathway enrichment analyses

Differential gene expression was assessed using the DESeq2 package in R (22) after filtering 6 samples that did not pass quality control because they had RIN scores less than 7.0 or were outliers as judged by principal component analysis and/or hierarchal clustering. The normal shrinkage estimator was employed to moderate fold-change estimates.

Module enrichment analysis was performed by applying the Fast Gene Set Enrichment (FGSEA) algorithm (23) to gene ranks that were derived by multiplying two values produced by DESeq: -log10(P-value) and the sign of the log2(fold-change). The set of gene modules used was compiled from six sources: (1) the C2 Reactome modules (24) from MSigDB (25), (2) a compilation of immune signatures from Nirmal et al. (26), (3) a repertoire of blood transcriptome modules from Chaussabel et al. (27), (4) PBMC modules extracted from the tmod R package (28), (5) the Hallmark modules from MSigDB (25), and (6) ASC, plasma cell, glomerulus and tubulointerstitial gene sets (29–31).

Canonical pathways were extracted using Ingenuity Pathway Analysis software (Qiagen) (http://www.ingenuity.com) (32).

### 2.15 Cross-species comparison

Raw data from lupus datasets were obtained using the analysis match comparison function of Ingenuity Pathway Analysis (32) and using the GEO repository GSE72535 for discoid lupus skin, GSE32591 from LN dissected glomerulus and tubulointerstitium and GSE36700 for lupus synovium. We compared the Z scores between processed microarray DE analysis of the four human SLE tissue experiments and the DE analysis of the KZR-616 treated vs. vehicle treated NZB/W mice.

### 2.16 Phase I clinical trial of KZR−616 in healthy volunteers

KZR-616-001 was a Phase 1, randomized, single center, double-blind, placebo-controlled study to evaluate the safety, tolerability, and pharmacokinetics of KZR-616 in healthy volunteers over 18 years old and was conducted in Melbourne, Australia (ACTRN12616001040459). In the single ascending dose (SAD) portion of the study, 32 subjects were enrolled into 4 SC dose cohorts and were randomized 6:2, KZR-616 to placebo (33). Single ascending dose levels included 7.5 (Cohort 1a), 15 (Cohort 1b), 30 (Cohort 1c), and 60 mg (Cohort 1d). All subjects received a single injection of either KZR-616 or placebo per protocol and were followed for 7 days after dosing or until resolution of any adverse events. In the multiple ascending dose portion, Cohorts received 4 weekly administrations of KZR-616 or placebo per protocol and were followed for 7 days after the last dose. In Cohort 2d, subjects receiving weekly administration at 45 mg were assessed for pharmacodynamics after 1 dose and for a functional assay (cytokine release) after the last dose. 

### 2.17 Proteasome inhibition pharmacodynamic assays

In the KZR-616-001 study, whole blood samples were collected at pre-dose and 4 hours after dosing in sodium heparin anticoagulant for determination of direct proteasome inhibition. Whole blood and isolated PBMCs (isolated by Ficoll gradient) were assessed for proteasome inhibition using two assays as described previously (34). Briefly, proteasome chymotrypsin-like activity was measured *via* fluorogenic succinyl-Leu-Leu-Val-Tyr-7-amino-4-methylcoumarin (Suc-LLVY-AMC) enzymatic assay in samples taken prior to and 4 hours after a single dose of KZR-616. Proteasome active site binding by KZR-616 was measured *via* Proteasome Constitutive Immuno-Subunit ELISA (ProCISE) in the same samples for all 6 subunits (constitutive proteasome β1, β2, β5; immunoproteasome LMP2, MECL-1, and LMP7), with values for only β5, LMP7, and LMP2 presented for brevity. For both proteasome inhibition assays, post-dose activity was normalized to pre-dose values.

### 2.18 Proteasome inhibition functional assay

In the KZR-616-001 study, whole blood samples with sodium heparin anticoagulant were collected at pre-dose and 4 hours post-dose in order to determine if KZR-616 could impact cytokine production. Whole blood was stimulated *ex vivo* overnight with PHA (1 µg/mL) or LPS (1 µg/mL). Supernatants were analyzed for cytokines *via* MSD multiplexed immunoassay (Meso Scale Diagnostics).

### 2.19 Statistical analysis

Statistical analysis was performed using Prism v6.0 (GraphPad). Comparisons for each pair were performed using a 2-tailed Student’s t test with Welch’s correction; multiple comparisons with a single control were performed using one-way ANOVA with Dunnett’s correction or Mann-Whitney.

### 2.20 Study approval

All animal studies were conducted in compliance with the NIH Guide for the Care and Use of Laboratory Animals and approved by Kezar Life Sciences Institutional Animal Care and Use Committee. Clinical trials were conducted in accordance with the principles of the Declaration of Helsinki (Ethical Principles for Medical Research Involving Human Subjects), and with the NHMRC National Statement on Ethical Conduct in Human Research (2007, incorporating all updates as of May 2015). For human studies, written informed consent was received prior to participation.

## 3 Results

### 3.1 Selective inhibition of the immunoproteasome blocks cytokine production in PBMCs from healthy donors and SLE patients

**Figure 1:**
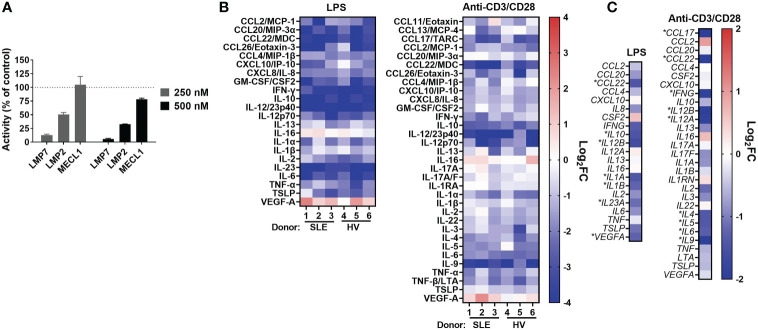
KZR-616 selectively inhibits the immunoproteasome and blocks cytokine production in human PBMCs. **(A)** Proteasome active site analysis following KZR-616 exposure for 1 hour in PBMCs from healthy volunteers (HV) using ProCISE. Post-KZR-616 treatment specific activity was normalized to DMSO vehicle control and is presented as mean (± SEM) relative activity. Representative of N=3 independent experiments are shown. **(B)** Cytokine production changes after 1 hour exposure of PBMCs to 500 nM KZR-616 followed by 24 hours stimulation with LPS or anti-CD3/anti-CD28 (N=3 donors). Values are expressed as log2 fold-change (log_2_FC) of KZR-616 treated relative to stimulated DMSO controls. Representative of N=3 independent experiments are shown. Only cytokines induced >5 fold in DMSO treated stimulated cells relative to unstimulated cells were included for the analysis. **(C)** Gene expression changes in PBMC from HV (2 donors) treated for 1h with 500 nM KZR-616 and stimulated for 20 hours. Values are expressed as log_2_FC relative to stimulated DMSO vehicle control. * indicates genes with significant changes: log_2_FC>1, Padj<0.01.

As part of the discovery of KZR-616, compound profiling was performed using cell free and cell-based systems that did not enable direct correlation of target inhibition to biologic outcomes such as cytokine production (17). The target inhibition profile of KZR-616 in human PBMCs was assessed following exposure for one hour at concentrations of 250 and 500 nM. At both concentrations, KZR-616 treatment resulted in near complete inhibition of LMP7 and >50% inhibition of LMP2, with minimal impact on MECL-1 activity (**Figure 1A**). These results are consistent with inhibition levels previously observed in MOLT-4 cells, which express approximately equivalent levels of both the constitutive proteasome and immunoproteasome and demonstrate that KZR-616 can mediate equivalent levels of target inhibition in cells regardless of cellular proteasome content (17).

Our previous report showed that KZR-616 inhibited production of 4 cytokines (IL-6, TNF-α, IL-12/IL-23p40, and IFN-γ) in healthy human PBMCs stimulated with either lipopolysaccharide (LPS) or anti-CD3 and anti-CD28 antibodies (17). Further assessment of the cytokine inhibitory potential of KZR-616 was done in PBMCs derived from both healthy volunteers and patients with SLE with matching of sex, age, and ethnicity (Demographic data in **Table S1**). Protein levels of 44 cytokines and chemokines (including two IL-17A tests) were evaluated to better understand the global effects of KZR-616 on leukocyte activation under 4 stimulation conditions targeting different immune cells and pathways: LPS, antibodies against CD3 and CD28, phytohemagglutinin (PHA), and phorbol 12-myristate 13-acetate (PMA) plus ionomycin. Of the 44 cytokines assessed, we observed that 37 were found to increase at least 5-fold in stimulated versus unstimulated cells in at least one stimulation condition (**Supplemental Figure 2B**). Pretreatment of cells with KZR-616 prior to stimulation resulted in cytokine inhibition under the LPS, PHA, and anti-CD3 and anti-CD28 stimulation conditions, but showed only a modest effect in cells stimulated with PMA and ionomycin (**Figure 1B** and **Supplemental Figures 2B**–**D**). Inhibition of >50% was observed for 19 cytokines and an additional 15 showed a significant decrease in KZR-616 treated cells versus untreated cells. Interestingly, the levels of 2 cytokines (VEGF and IL-16) were increased in KZR-616 treated samples. The profile of cytokine release modulation, with respect to both the specific cytokines affected and the level of inhibition was similar in LPS and anti-CD3/CD28 stimulated PBMCs derived from healthy volunteers and patients with SLE (**Figure 1B**).

To better understand the effects on cytokine secretion and immune cell activation, global gene expression was assessed by RNA-Seq in PBMCs from healthy volunteers that were treated with 500 nM of KZR-616 and then stimulated with either LPS or antibodies against CD3 and CD28 *in vitro* (**Figure 1C**). Differential expression (DE) analysis of LPS stimulated PBMCs identified 546 genes that were significantly modulated following exposure to KZR-616 (adjusted P-value < 0.01 and fold-change ≥ 2). Of these, 60 genes were significantly increased, and 486 genes were decreased in the KZR-616-treated group compared to controls. Of the 18 cytokines for which inhibition was detected at the protein level under this stimulation condition, 6 cytokines showed a significant reduction in mRNA levels. LPS acts as an endotoxin *via* its binding of the CD14/TLR4/MD2 receptor complex in monocytes, dendritic cells, macrophages and B cells, which promotes the secretion of pro-inflammatory cytokines (35). Decreases in TLR2, TLR4, CD14 and MMP9 were observed, supporting a direct down-regulating effect on TLR signaling (data not shown). Similarly, DE analysis of PBMCs stimulated through the T cell receptor (TCR) and CD28 revealed that 270 genes were differentially expressed between the KZR-616-treated and untreated samples. Among these, 154 genes were up-regulated, and 116 genes were down-regulated in the KZR-616-treated group compared to controls. Of the 26 genes encoding cytokines for which inhibition was observed at the protein level, 9 were significantly downregulated. Interestingly, VEGFA, which was increased at the protein level, trended downward at the gene level. The discrepancy of protein level and gene expression level may indicate differences in kinetics between the two. Taken together, these findings demonstrate that KZR-616 blocks both mRNA and protein production of a wide range of cytokines in activated leukocytes from both healthy individuals and patients with SLE, likely *via* reduced gene expression.

### 3.2 KZR-616 affects T and B lymphocyte differentiation

**Figure 2:**
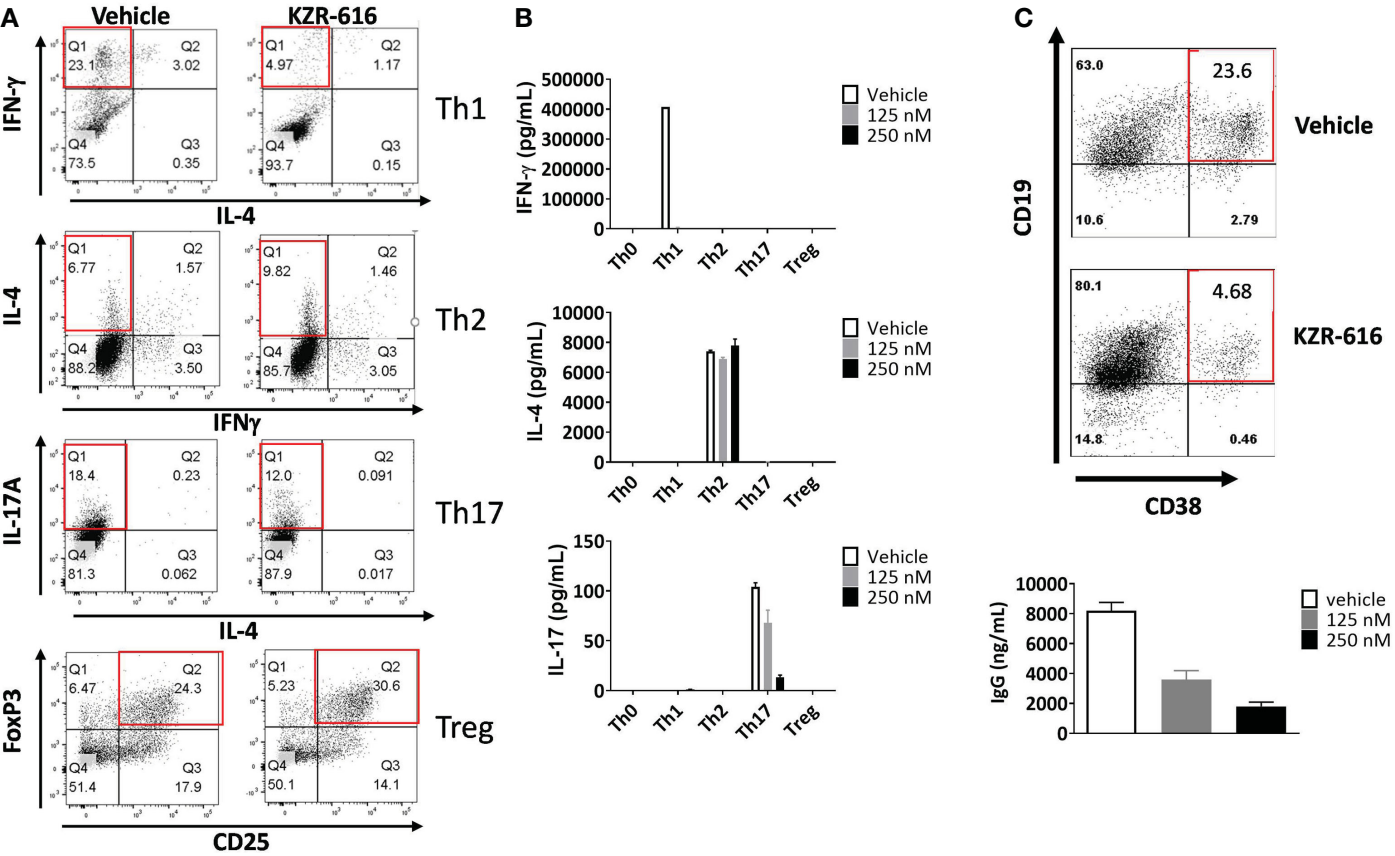
KZR-616 inhibits human T helper cell and B cell plasmablast differentiation. **(A)** Naïve CD4^+^ T cells were treated with DMSO vehicle or 250 nM KZR-616 as a 1 hour pulse prior to stimulation and differentiation to Th1, Th2, Th17 and Treg cells under polarizing culture conditions and intracellular cytokines and FoxP3 transcriptional factor were monitored. **(B)** Cytokine secretion from differentiated T cells treated with DMSO or KZR-616 at 125 or 250 nM was monitored from tissue culture supernatants. Representative of N=3 independent experiments. **(C)** Human peripheral blood CD19^+^ B cells were treated with DMSO vehicle or KZR-616 as a 1 hour pulse and stimulated *in vitro* with anti-CD40 and anti-IgM antibodies in the presence of IL-21 for 6 days. Plasmablasts were confirmed by flow cytometry analysis for CD38 expression and secretion of IgG into culture media. Representative of N=3 independent sets of experiments are shown in **(A–C)**.

Inhibition of the immunoproteasome with ONX 0914 has been shown to block the formation of Th1 and Th17 cells from mouse splenocytes and increase the number of regulatory T cells *in vitro* and *in vivo* (14–16). To address the effect of KZR-616 on human T cell polarization, human naïve CD4^+^ T cells were stimulated under differentiating conditions to generate Th1, Th2, Th17 and Treg cells. Inhibition of the immunoproteasome resulted in reduced Th1 (IFN-γ ^+^) and Th17 (IL-17^+^) polarization as assessed by intracellular cytokine staining (ICS) and direct cytokine measurement in cell culture media (**Figures 2A, B**). We also noted a modest increase in the number of Foxp3^+^ Treg cells following exposure to KZR-616. In contrast, no effects on the number of IL-4^+^ cells or secretion of IL-4 under Th2-polarizing conditions were seen with KZR-616 treatment.

Previous work has demonstrated a reduction in plasma cells in lupus prone mice and rats receiving allografts with ONX 914 treatment indicating that immunoproteasome inhibition may have direct effect on B cell differentiation (13, 36). We stimulated human peripheral blood CD19^+^ B cells *in vitro* with anti-CD40 and anti-IgM antibodies in the presence of IL-21 for 6 days, which resulted in robust differentiation into CD19^+^CD38^+^ IgG secreting plasmablasts (**Figure 2C**). KZR-616 treatment prior to stimulation significantly reduced the number of CD19^+^CD38^+^ plasmablasts as well as IgG levels in culture supernatants. Interestingly, we found no effect on IgG secretion from pre-differentiated plasmablasts, indicating that KZR-616 may be blocking differentiation rather than directly impacting IgG production from plasma cells (**Supplemental Figure 3**).

### 3.3 Selective inhibition of the immunoproteasome with KZR-616 ameliorates nephritis progression in lupus-prone mice

**Figure 3:**
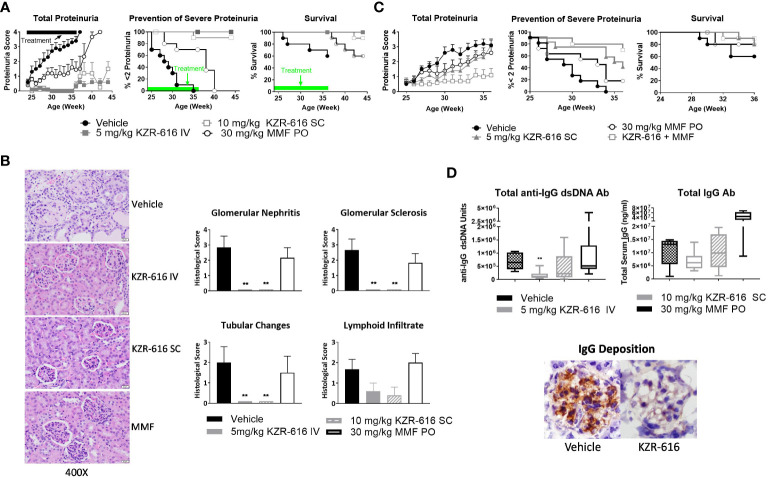
KZR-616 ameliorates nephritis progression in lupus-prone mice. **(A)** NZB/W F1 mice (proteinuria grade 1 at study start) were treated QODx3 with vehicle (IV) or KZR-616 (5 mg/kg IV or 10 mg/kg SC) or daily with MMF (30 mg/kg PO) for 13 weeks followed by an 8-week non-dosing period for KZR-616 and MMF groups (n=10/group). **(B)** Representative kidney sections following 13 weeks of treatment. Histological changes were scored from 0-4 for glomerular nephritis, sclerosis, tubular changes and lymphoid infiltrates. **(C)** NZB/W F1 mice (proteinuria grade 1 at study start) were treated QODx3 with vehicle (IV) or KZR-616 (5 mg/kg SC), daily with MMF (30 mg/kg PO), or the combination of KZR-616 and MMF for 12 weeks (n=10/group). **(D)** Serum collected after 12 weeks of treatment as described in A was assessed for anti-dsDNA IgG and total IgG levels. IgG deposition in representative kidney sections of vehicle vs. KZR-616 treated animals. Group means ± SEMs were calculated for each group; quantitative group means were compared with vehicle using one-way ANOVA with Dunnett’s correction, **P < 0.01 **(B, D)**.

Proteasome inhibition with agents such as bortezomib and ONX 0914 has been shown to block the progression of lupus nephritis in mouse models, and bortezomib has successfully been applied to patients with SLE and LN, resulting in symptom improvement (13, 37, 38). Therefore, we evaluated the ability of KZR-616 to inhibit progression of lupus nephritis in the NZB/W F1 mouse model of lupus and to probe the durability of the amelioration of disease progression. Thrice weekly (QODx3) intravenous (IV) or subcutaneous (SC) administration of KZR-616 to NZB/W F1 mice with established nephritis resulted in blocking the progression of disease as measured by proteinuria (**Figure 3A**). Upon cessation of treatment, mild nephritis developed during the 8 weeks of follow-up after the last dose, but no animals progressed to severe proteinuria as had previously occurred in all vehicle animals. Additionally, once weekly (QW) IV administration of KZR-616 at 10 mg/kg achieved a similar level of proteinuria reduction compared with the QODx3 schedule (**Supplemental Figure 4**). In contrast, daily oral administration of 30 mg/kg MMF reduced disease progression to a lesser degree than KZR-616 and upon cessation of treatment, severe proteinuria rapidly developed. In addition, prolonged survival in animals treated with KZR-616 compared to either the vehicle control or MMF-treated mice was observed (**Figure 3A**). KZR-616 treated animals showed a nearly complete absence of glomerular nephritis and tubular changes in the kidneys (**Figure 3B**). Next, we administered KZR-616 to MRL/lpr mice with active proteinuria and monitored for proteasome active site occupancy. Similar to our prior report (13), we found that KZR-616 administration resulted in selective inhibition of LMP7 and LMP2 in mouse tissues (**Supplemental Figure 5**). Treatment of MRL/lpr mice with KZR-616 improved both proteinuria and skin lesions relative to vehicle control demonstrating that responses may be independent of the genetic background of the animals (**Supplemental Figure 5**).

### 3.4 KZR-616 combines effectively with MMF to attenuate LN in NZB/W F1 mice

Given that MMF is a standard of care in the treatment of LN, we investigated the potential benefit of a combination of KZR-616 and MMF in diseased mice. We chose a QW SC dose level of 5 mg/kg, half the dose used in the single agent treatment described above to achieve a suboptimal therapeutic response to KZR-616. In comparison with vehicle-treated mice and animals treated with either agent alone, the combination of MMF and KZR-616 markedly reduced the levels of proteinuria and increased survival in diseased animals (**Figure 3C**).

### 3.5 KZR-616 treatment decreases anti-dsDNA IgG and total IgG levels in NZB/W F1 mice

Evaluation of serum samples taken from mice after 13 weeks of treatment with KZR-616 showed that anti-dsDNA IgG levels were significantly reduced, and total IgG levels were mildly but not significantly decreased compared to vehicle-treated animals (**Figure 3D**). The reduction in serum IgG levels correlated with reduced levels of total IgG deposition in the glomeruli as compared to vehicle-treated mice (**Figure 3D**). In contrast, no effect was observed with MMF treatment on anti-IgG dsDNA or total IgG levels. To further examine the effects of KZR-616 on immunoglobulin production, we administered the T cell-dependent antigen, keyhole limpet hemocyanin (KLH), to mice treated with KZR-616 and followed their IgM and IgG responses. No significant changes in anti-KLH antibodies were observed following KZR-616 treatment (**Supplemental Figure 6A**). Similar findings were seen in monkeys receiving up to 9 months of weekly treatment with KZR-616 and immunized with KLH (**Supplemental Figure 6B**). Taken together, these data suggest that KZR-616 can modulate pathogenic (i.e., autoimmune) responses without affecting B cell responses to foreign antigens.

### 3.6 Immunophenotypic analysis of KZR-616 in lupus prone mice

**Figure 4:**
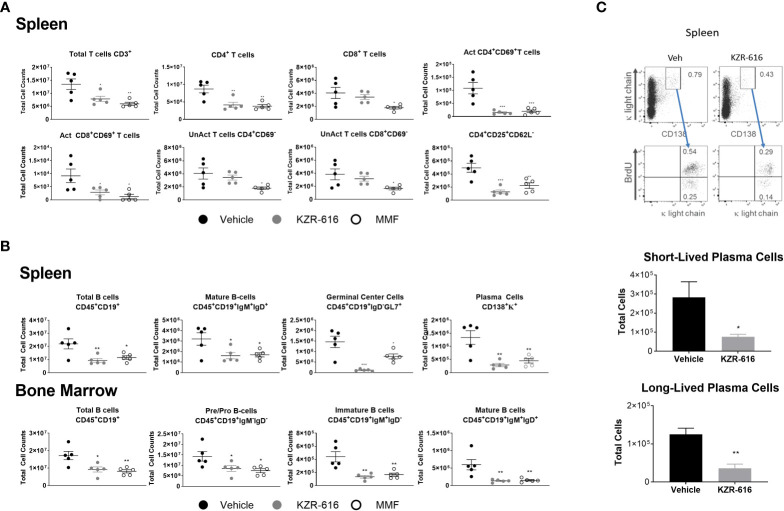
KZR-616 reduces the number of T, B and plasma cell populations in NZB/W F1 lupus mice. Flow cytometric analysis was performed on the spleen and bone marrow from mice treated as in **Figure 3A**. **(A)** Numbers of total CD3^+^ T cells, CD4^+^ T cells, CD8^+^ T cells, CD4^+^ CD69^+^ activated T cells, CD8^+^ CD69^+^ activated T cells, CD4^+^CD69^-^ unactivated T cells, CD8^+^CD69^-^ unactivated T cells and CD4^+^CD25^+^CD62L^-^ regulatory T cells in spleen (N=5). **(B)** Numbers of total CD45^+^CD19^+^ B cells, CD45^+^CD19^+^IgM^+^IgD^+^ mature B cells, CD45^+^CD19^+^IgD^-^GL7^+^ germinal center B cells, and CD138^+^κ^+^plasma cells in the spleen. Numbers of total CD45^+^CD19^+^ B cells, CD45^+^CD19^+^IgM^-^IgD^-^ pre/pro B cells, CD45^+^CD19^+^IgM^+^IgD^-^ immature B cells, and CD45^+^CD19^+^IgM^+^IgD^+^ mature B cells in the bone marrow (N=5). **(C)** Representative flow cytometric analyses of short- and long-lived plasma cells in spleen from NZB/W F1 mice treated for 2 weeks with vehicle or KZR-616 (N=5) (left panel). The gated plasma cells expressing CD138 and cytoplasmic κ-light chain expression were discriminated as BrdU^+^ short-lived and BrdU^-^ long-lived plasma cells (bottom panel). Numbers in the top panels represents percentages of plasma cells with respect to total cell number. Bar graphs of flow cytometric analyses of the total CD138^+^ cytoplasmic κ^+^ BrdU^+^ short-lived and CD138+ cytoplasmic κ^+^ BrdU^-^ long-lived plasma cells in the spleens of KZR-616-treated and control mice. Statistical significance was determined using Student's t-test between vehicle and treatment groups *P < 0.05, **P < 0.01, *** P < 0.001 **(A, B)**.and unpaired student’s *P < 0.05, **P < 0.005 **(C)**.

Previous studies in both SLE patients and lupus-prone mice have demonstrated abnormalities in the function, distribution, and gene-expression patterns of various T cell subsets, as well as increased plasma cell activity (39–41). We evaluated splenic T cell populations and both splenic and bone marrow B cell populations by flow cytometry following 13 weeks of treatment in NZB/W F1 mice. Treatment with either KZR-616 or MMF resulted in a decrease in total CD4^+^ T cells, but KZR-616 did not impact CD8^+^ T cell numbers (**Figure 4A**). Both agents were associated with reduced numbers of activated (CD4^+^CD69^+^ and CD8^+^CD69^+^) T cells in the spleen (**Figure 4A**). Treatment with MMF, but not KZR-616 resulted in depletion of inactive (CD4^+^ CD69^-^ and CD8^+^ CD69^-^) T cells, consistent with the action of MMF as an inhibitor of DNA replication (42). Treatment with KZR-616 did not impact CD8^+^ T cell numbers and both agents were associated with a significant decrease in the number of regulatory T cells (CD4^+^CD25^+^CD62L^-^) (**Figure 4A**). In both spleen and bone marrow, CD19^+^ B cell numbers were reduced following treatment with KZR-616 (**Figure 4B**). Both KZR-616 and MMF significantly decreased early-stage B cells (pro- and pre-B cells), as well as immature and mature B cells in the bone marrow (**Figure 4B**). However, KZR-616 was associated with a more pronounced impact on splenic germinal center B cells compared to MMF. In addition, plasma cells (CD138^+^κ^+^) were decreased in KZR-616 treated animals by 63% and 79% in the bone marrow (data not shown) and spleen, respectively (**Figure 4B**). Enzyme-linked immunospot (ELISPOT) assays further demonstrated that both anti-dsDNA IgG and total IgG antibody secreting cells (ASCs) in both the spleen and bone marrow were reduced following KZR-616 treatment (**Supplemental Figure 7**). To further evaluate the effects of KZR-616 on plasma cells, diseased mice treated with either vehicle or KZR-616 were exposed to BrdU in the drinking water for 2 weeks to enable quantitation of proliferating cells. Total plasma cells (CD138^+^, intracellular κ^+^) were significantly reduced following KZR-616 treatment, and this included similar relative reductions in both short-lived (CD138^+^, intracellular κ^+^, BrdU^+^) and long-lived (CD138^+^, intracellular κ^+^, BrdU^-^) plasma cells (**Figure 4C**). Taken together, these data suggest that KZR-616 can impact both T cell activation and B cell function in diseased animals, leading to reductions in pathogenic lymphocyte responses.

### 3.7 Immunoproteasome inhibition decreases mRNA expression of multiple immune signaling pathways and B cell signatures in the spleen

**Figure 5:**
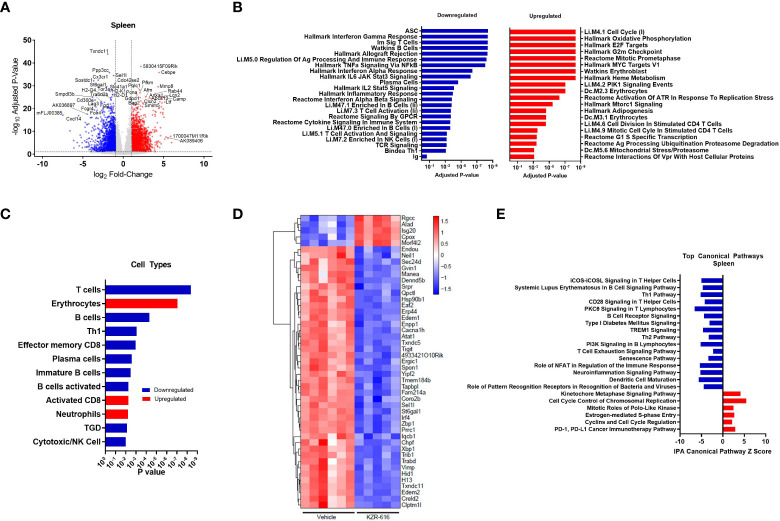
Global gene expression and pathway changes in spleen following KZR-616 treatment in NZB/W F1 Mice. NZB/W mice (proteinuria grade 1) were treated with vehicle QODx3 or 5 mg/kg IV KZR-616 QODx3 for 13 weeks. RNA sequencing was performed on spleens (n=5-6/group). **(A)** Volcano plot of the differential expression of KZR-616 vs vehicle-treated spleens. **(B)** The most significantly depleted and enriched modules as determined by FGSEA of the gene-level differential expression results. **(C)** The subset of the most significantly depleted and enriched modules that are signatures of cell types [Nirmal 2018]. **(D)** Row-scaled heatmap of expression at the sample level for the most significantly differentially expressed (P < 10^-8^) ASC-specific genes. Each column represents one animal. **(E)** The most significantly decreased and increased pathways by Z score analysis with an overlap p-value > 0.05 as determined by Ingenuity Pathway Analysis.

To more fully characterize the phenotypic changes observed in the lymphocyte compartments of NZB/W mice at the molecular level, we utilized RNA-Seq to profile the transcriptomes of whole spleens from mice that were treated for 13 weeks with either vehicle or KZR-616. Spleen samples were also collected from untreated, pre-nephritic mice (10 weeks of age) to assess gene expression changes associated with disease progression. We found 412 genes were differentially expressed (228 upregulated and 184 downregulated) in the spleens of diseased mice compared to pre-nephritic animals (adjusted P-value < 0.01 and fold-change ≥ 2). A total of 3,065 genes were differentially expressed (1,597 upregulated and 1,468 downregulated) in the spleens of KZR-616-treated mice compared to vehicle-treated mice (**Figure 5A** and **Table S2**).

We applied fast gene set expression analysis (FGSEA) to the differential expression results to identify biological pathways and cell types affected by KZR-616 (23). As a result of treatment with KZR-616, we observed a decrease in the expression of gene modules related to adaptive and innate immune signaling processes, including signaling by multiple cytokines (IFN-α, IFN-γ, IL-2, IL-6 and TNF-α), GPCR signaling, inflammatory response, and antigen presentation (**Figure 5B** and **Table S3**). Consistent with our other *in vivo* findings, KZR-616 administration resulted in decreased expression of many immune cell type modules, including T cells [Th1, γδT (TGD), and effector memory CD8], B cells (immature and activated) and plasma cells (**Figure 5C** and **Table S2**). However, in contrast to our flow cytometry findings, gene expression analysis indicated a modest increase in the expression of the activated CD8 module in the spleen. Expression of the immunoproteasome subunits Psmb8 (LMP7), Psmb9 (LMP2) and Psmb10 (MECL-1) was significantly reduced following treatment with KZR-616, possibly reflecting the overall anti-inflammatory activity observed in these animals (**Table S2**). To assess the plasma cell compartment, we included two previously identified modules associated with ASCs and plasma cell differentiation in our gene module analysis (29, 30). Of particular note, the ASC gene set was the most significantly down-regulated of any gene set included in our analysis (adjusted P-value ≤ 10^-8^) (**Figure 5B**). 75 of 275 genes in the ASC module with detectable expression were differentially expressed between the spleens of KZR-616- and vehicle-treated mice. A clustergram highlighting the subset of the most significantly differentially expressed (adjusted P-value ≤ 10^-8^) genes in the module is shown in **Figure 5D** (full clustergram available in **Supplemental Figure 8**). Further analyses of these ASC-specific transcripts that were induced (fold change > 2, *P* < 0.01) in vehicle-treated compared with naive animals showed that KZR-616 normalized expression of the majority of them including Blimp (Prdm1), Il21, Xbp1, and Irf4 (**Table S2**). These findings in gene expression changes with KZR-616 treatment are in agreement with *in vivo* observations of reduction of plasma cells and IgG. In addition, gene modules associated with erythrocytes and neutrophils, heme metabolism, cell cycle and oxidative phosphorylation, were upregulated in mice after KZR-616 treatment and may represent a normalization of the compensated hemolytic anemia that occurs in NZB/W mice (43) (**Figure 5B** and **Table S3**).

Ingenuity Pathway Analysis (IPA) was employed to further investigate the specific pathways affected by immunoproteasome inhibition. The top 22 decreased and increased pathways detected by IPA in the spleen following KZR-616 treatment [-log (p-value) > 7.59] are listed in **Figure 5E** (**Table S4**). As with the results of FGSEA analysis, the predominant trend was down-regulation of innate and adaptive immune signaling pathways, including iCOS-iCOSL signaling in T helper cells, Th1 pathway, CD28 signaling in T helper cells, PKCθ signaling in T lymphocytes and TREM1 signaling. Consistent with our *in vitro* and *in vivo* observations of decreased B cell differentiation and plasma cells, KZR-616 reduced multiple B cell signaling pathways, including B cell receptor signaling, PI3K signaling in B lymphocytes and the B cell signaling pathway in SLE. Some of the genes most significantly downregulated in the latter module included Il21, Irf5, Irf7, Ap1, Nfatc1, Nfkb1, Nfkb2, Stat1, Stat2 and Stat3, which are known to be important for B cell development, proliferation and survival, germinal center formation, and antibody production (full list of genes in **Table S4**). We also identified 5 upregulated pathways: the kinetochore metaphase signaling pathway, cell cycle control of chromosomal replication, PD-1/PD-L1 cancer immunotherapy pathway, mitotic roles of polo-like kinase, estrogen-mediated S-phase entry, and cyclins and cell cycle regulation.

### 3.8 Immunoproteasome inhibition reduces the expression of multiple signaling pathways and genes associated with human lupus nephritis in the kidney

**Figure 6:**
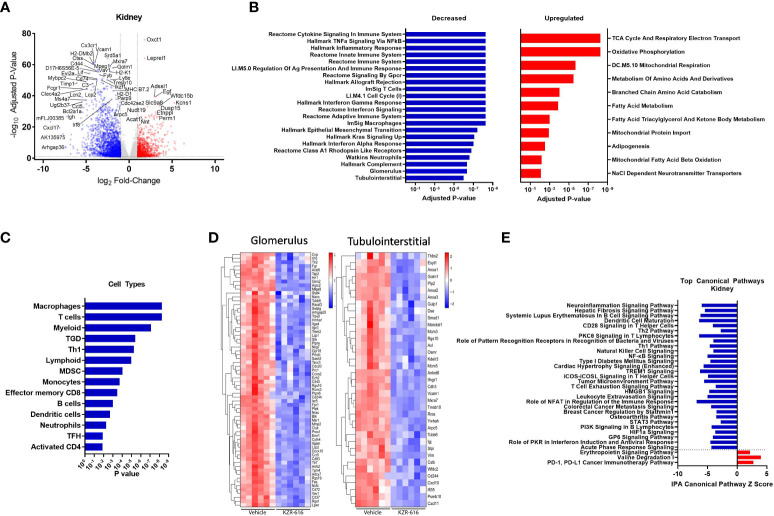
Global gene expression and pathway changes in kidney following KZR-616 treatment in NZB/W F1 mice. RNA sequencing was performed on kidneys (n=5-6/group) derived from the experiment described in **Figure 5**. **(A)** Volcano plot of the differential expression of KZR-616 vs vehicle-treated kidneys. **(B)** The most significantly depleted and enriched modules as determined by FGSEA analysis of the gene-level differential expression results. **(C)** The subset of the most significantly depleted and enriched modules that are signatures of cell types [Nirmal 2018]. **(D)** Row-scaled heatmaps of expression at the sample level for the most significantly differentially expressed (P < 10^-5^) glomerular and tubulointerstitial-specific genes. **(E)** The most significantly decreased and increased pathways by Z score analysis with an overlap p-value > 0.05 as determined by Ingenuity Pathway Analysis.

Whole transcriptome profiling was also performed on kidneys taken from vehicle- and KZR-616-treated mice to determine the effects of immunoproteasome inhibition on both immunologic and tissue remodeling in what is the main target tissue of disease in NZB/W F1 mice. Kidney samples were also collected from untreated, pre-nephritic mice (10 weeks of age) to assess gene expression changes induced during disease progression. We found 2,909 genes were differentially expressed (1,864 upregulated and 1,045 downregulated) in the kidneys of diseased mice compared to pre-nephritic animals (adjusted P-value < 0.01 and fold-change ≥ 2). A total of 2,633 genes were differentially expressed (773 upregulated and 1,860 downregulated) in the kidneys of KZR-616-treated mice compared to vehicle-treated animals (**Figure 6A** and **Table S2**).

As with the spleen data, FGSEA of kidney gene expression revealed that KZR-616 treatment preferentially reduced levels of modules associated with inflammatory cytokine signaling and response (TNF-α, IFN-γ and IFN-α), innate and adaptive immune response, cell cycle and cellular migration (**Figure 6B**). These same modules were also upregulated in vehicle animals when compared to pre-nephritic/pre-diseased animals (**Table S3**). Pathways with increased expression due to KZR-616 were primarily associated with mitochondrial transport, metabolism, and oxidative phosphorylation, all of which were downregulated in vehicle-treated nephritic mice relative to pre-nephritic animals (**Figure 6B** and **Table S3**). Consistent with our histological findings of decreased cellular infiltration in the kidney, KZR-616 administration resulted in reduced expression of multiple myeloid, T, and B cell gene signatures (**Figure 6C** and **Table S3**). Of note, genes encoding Cxcl17, Ig heavy chain (Igh) and IL-36 (Il1f6) were amongst the top 8 most down-regulated. In addition, KZR-616 decreased expression of the genes encoding lipocalin 2 (Lcn2), and fibronectin (Fn1), which are markers of proximal tubule damage (31). Berthier et al. previously reported gene expression changes in kidneys of 32 LN patients relative to 15 healthy living donors (31). We assessed the expression of the mouse orthologs of genes known to be specifically dysregulated in the glomerulus and tubulointerstitial compartments of patients with active nephritis and found that 117 of these 180 genes were also upregulated in vehicle-treated diseased mice compared to pre-nephritic animals (**Table S2**). Of these 117 genes, 93 were significantly downregulated in the kidneys following KZR-616 treatment (**Figure 6D**). As with LN patients, where kidneys show increased levels of Psmb10 (MECL-1) and Psmb8 (LMP7) relative to kidneys from healthy individuals (11, 31), we found significantly increased expression of all 3 immunoproteasome subunits (LMP7, LMP2, and MECL-1) in kidneys derived from diseased mice treated with vehicle compared to pre-nephritic animals (**Table S2**). The expression of these subunits was significantly reduced following treatment with KZR-616, likely reflecting the overall anti-inflammatory activity observed in this study.

IPA further delineated the specific innate and adaptive immune signaling, cell migration and tissue remodeling pathways that were affected with KZR-616 treatment, many of which were also found in the spleen. In addition, pathways involved in hepatic fibrosis, roles of pattern recognition receptor in recognition of bacteria and viruses, NK cell, and T cell exhaustion were also found to be down-regulated following KZR-616 treatment (**Figure 6E**). Many of these canonical pathways have been previously shown to be upregulated in nephritic kidneys of NZB/W F1 mice as well as in human lupus (31). In contrast, only 3 pathways were upregulated in the top 22 pathways affected by KZR-616: erythropoietin signaling, valine degradation, and PD-1/PD-L1 cancer immunotherapy.

### 3.9 Comparison of enriched pathways shared between blood, spleen and kidney of NZB/W F1 mice following KZR-616 treatment

**Figure 7:**
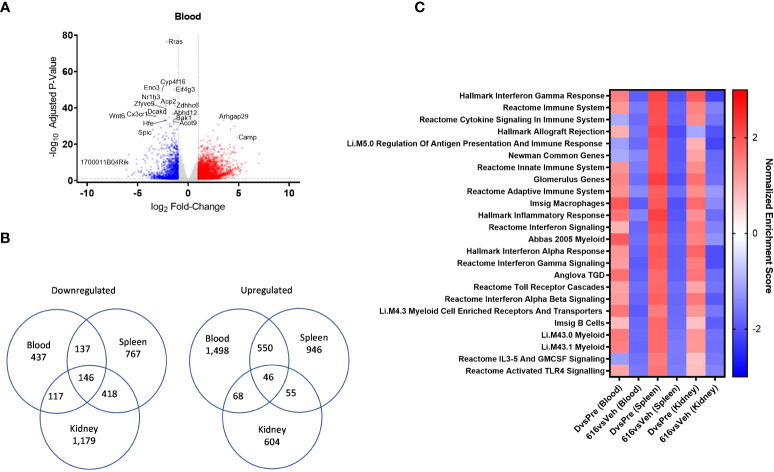
Comparative pathway analysis of blood, spleen, and kidney from NZB/W F1 mice following KZR-616 treatment. RNA sequencing was performed on blood, spleen, and kidney (n=5-6/group) derived from the experiment described in **Figure 5**. **(A)** Volcano plot of the differential expression of KZR-616 vs vehicle-treated blood. **(B)** Venn diagrams of genes significantly downregulated (left) or upregulated (right) in blood, spleen and kidney. **(C)** Heatmap of normalized enrichment scores for modules that were significantly depleted after KZR-616 treatment in blood, spleen, and kidneys sorted on p value. The enrichment scores for vehicle-treated diseased vs pre-disease (10-week-old) blood, spleen, and kidneys are also shown.

As part of the ongoing clinical evaluation of KZR-616, it would be useful to know whether the molecular changes described above are also occurring in patients. Since kidney biopsies put patients at an undue risk, we sought to understand whether gene expression changes in blood mirrored those seen in the spleen and kidney. We analyzed the gene expression in the blood of diseased mice and compared to pre-nephritic animals as well as across tissues (spleen and kidney). We found 2,511 genes were differentially expressed (1,648 upregulated and 863 downregulated) in the blood of diseased mice compared to pre-nephritic animals (adjusted P-value < 0.01 and fold-change ≥ 2). Overall, 2,999 genes were differentially expressed (2,162 increased and 837 decreased) in whole blood following KZR-616 treatment (**Figure 7A** and **Table S2**). We found that only a small fraction of differentially expressed genes is shared between any two tissue types (**Figure 7B**). For example, only 14% of the 1,860 genes that were significantly downregulated after KZR-616 treatment in kidney were also downregulated in blood. Across all 3 tissue types, 146 genes were downregulated and 46 genes upregulated (**Figure 7B**).

We applied FGSEA to identify which gene modules were differentially expressed in blood and to compare those with modules that were previously recognized in the spleen and kidney. Of the 1,139 modules analyzed, 30% (344) were downregulated in at least one tissue following KZR-616 treatment (padj ≤ 0.01). Of these 344 modules, 24 were downregulated in all 3 tissue types (**Figure 7C**). Amongst those modules were pathways highlighted above, including IFN-γ, IFN-α and signatures of B, γδT cells, and myeloid cells. Down-regulation of Toll-like receptors and IL-3/IL-5/GM-CSF signaling were also shared across blood, spleen, and kidney. Correspondingly, these modules were upregulated across all 3 tissue types in diseased animals relative to their pre-disease counterparts (**Figure 7C** and **Table S5**). Taken together, these data suggest that gene expression profiling from whole blood in mice captures molecular changes occurring within lymphoid and disease target tissues which may be mechanistically relevant and that whole blood might be used as a surrogate in human patients.

### 3.10 KZR-616-mediated gene expression changes relate to disease-specific alterations in patients with active SLE

To further explore the clinical relevance of immunoproteasome inhibitor-mediated suppression of molecular pathways in lupus-prone mice, we compared gene expression profiles in spleen and kidney from KZR-616-treated mice with profiles in skin, synovium, and kidney (glomerulus and tubulointerstitium) derived from SLE patient samples using the IPA “Analysis Match” feature. Analysis of the human tissues revealed activation of the majority of the top 30 canonical pathways shared across all four tissues. The top five canonical pathways, all of which have previously been shown to be upregulated in both nephritic mice and human LN patient tissues, were role of nuclear factor activated T cells (NFAT) in regulation of immune response, PKθ signaling in T lymphocytes, dendritic cell maturation, SLE in B cell signaling pathway, and crosstalk between dendritic cells and natural killer cells (**Supplemental Figure 9** and **Table S6**). In contrast, the molecular pathways that were highly upregulated in human lupus-affected organs were decreased by KZR-616 in the spleen or kidney of treated NZB/W F1 mice, further supporting the broad effects of immunoproteasome inhibition on the immune system and the therapeutic potential in human SLE.

### 3.11 KZR-616 induces selective inhibition of the immunoproteasome and blocks immune effector cell function in healthy volunteers

**Figure 8:**
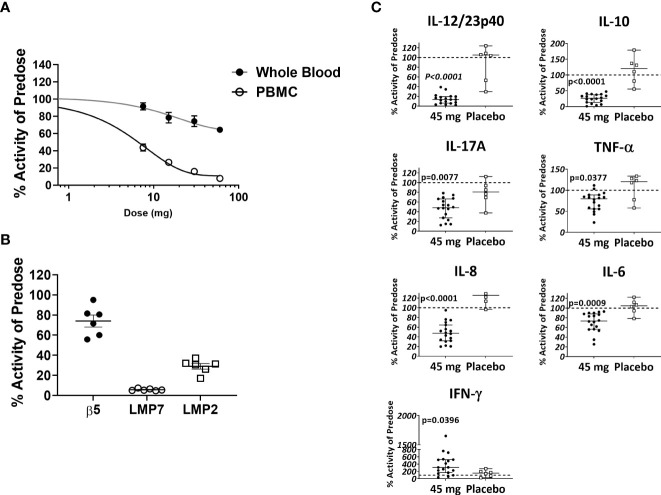
KZR-616 administration to healthy volunteers induces selective inhibition of the immunoproteasome and blocks *ex vivo* stimulated cytokine release. **(A)** Proteasome chymotrypsin-like activity was measured prior to and 4 hours after a single dose of KZR-616 at 7.5, 15, 30, and 60 mg (n = 6/timepoint). Post-dose specific activity was normalized to pre-dose values and is presented as mean (± SEM) relative activity. **(B)** Proteasome subunit occupancy following a single dose of 45 mg was assessed using ProCISE, normalized to pre-dose values and is presented as individual subject values with mean ± SEM. **(C)** Whole blood was drawn from subjects prior to and 4 hours after administration of placebo or 45 mg KZR-616 and stimulated with LPS for 20 hours *in vitro*. Supernatants were analyzed for cytokines *via* multiplexed MSD immunoassay and normalized to pre-dose levels (dotted line). P values were derived from Mann-Whitney test.

Based in part on the experimental results presented here, KZR-616 is currently being studied in Phase 2 clinical trials in patients with LN (NCT# 03393013) and polymyositis or dermatomyositis (NCT# 04033926). Prior to initiation of these studies, KZR-616 was administered as a single dose or 4 weekly subcutaneous doses to healthy volunteers (males and females) at doses ranging from 7.5 to 60 mg (ACTRN12616001040459). Single and multiple dose administration of KZR-616 was generally well tolerated and a maximum tolerated dose was not reached in this study (Manuscript in preparation). Proteasome activity was measured in whole blood (predominantly constitutive proteasome) and purified PBMCs (predominantly immunoproteasome) taken 4 hours after dosing using an enzymatic assay for chymotrypsin-like activity [contributed by β5 (constitutive proteasome)/LMP7 (immunoproteasome) subunits] or *via* an active site occupancy assay described previously (34). In samples taken prior to dosing, we found no difference between specific activity levels in baseline PBMCs in males and females (**Supplemental Figure 10**). We observed a dose-dependent and selective inhibition of LMP7 activity following a single dose of KZR-616 (**Figure 8A**). Recovery of immunoproteasome activity was seen as early as 24 hours after dosing and was nearly complete by 7 days (data not shown). At a dose of 45 mg, mean inhibition of LMP7 and LMP2 was >90% and >70%, respectively, while inhibition of β5 was <35% (**Figure 8B**). After 4 weekly administrations of either placebo or KZR-616 at 45 mg, whole blood was drawn from subjects prior to and 4 hours after the last dose and exposed to LPS or PHA for 20 hours. Similar to what was observed in human PBMC exposed *in vitro* to KZR-616, we found that expression of several cytokines, including TNF-α, IL-17, the p40 subunit of IL-12/23, IL-6, and IL-10, were significantly reduced in subjects receiving KZR-616 but not in subjects receiving placebo (**Figure 8C** and **Supplemental Figure 11**). These results suggest that KZR-616 administration in humans results in a similar profile of selective immunoproteasome inhibition and anti-cytokine activity to that seen in preclinical studies.

## 4 Discussion

Based on their use as in the treatment of multiple myeloma, bortezomib and other agents that target multiple proteasome forms have been employed successfully in patients with refractory, B cell-driven autoimmune and immune-mediated disorders (44). Selective inhibition of the immunoproteasome with agents such as KZR-616 and ONX 0914 has been shown to yield anti-inflammatory activity similar to bortezomib and other proteasome inhibitors in mouse models of autoimmunity but is not directly cytotoxic to either transformed or non-transformed lymphocytes as has been reported with bortezomib (13, 15, 17, 45). Based on phenotypic and limited protein analysis, immunoproteasome inhibition has been demonstrated to impact multiple immune effector cell types including B cells, plasma cells, T cells, and dendritic cells (13–16, 36, 46). In the current study, we have dramatically expanded our mechanistic understanding of immunoproteasome inhibition using multiplexed cytokine analysis and global transcription profiling *in vitro* and in a mouse model of LN. These studies also represent the first assessment of global gene expression changes following treatment with a selective immunoproteasome inhibitor *in vivo*. Selective inhibition of the immunoproteasome broadly inhibits inflammatory cytokines at the gene expression and protein levels following TLR or TCR activation. It is noteworthy that effects on both type I and II interferon and TLR pathways were seen in human immune effector cells and in the blood of diseased mice treated with KZR-616, suggesting that these represent important mechanisms for the therapeutic response of this novel agent. Additionally, the large number of cytokines shown to be affected by KZR-616 suggests a more extensive role for the immunoproteasome in the regulation of immune responses than has previously been appreciated. Finally, KZR-616 blocked the differentiation of both human Th1 and Th17 cells but had no impact on Th2 cells, which is consistent with previous findings in mouse cells (14).

KZR-616 induced a profound and durable reduction in nephritis development in diseased NZB/W mice. In comparison to MMF, a standard of care in the treatment of LN, KZR-616 resulted in larger symptomatic (proteinuria), biomarker (anti-dsDNA antibodies), and histologic improvements. In addition, the rapid progression of disease in MMF-treated animals following cessation of treatment suggests that the mechanism of KZR-616 results in durable immunomodulation, rather than global and transient immunosuppression. This hypothesis is supported by the lack of KZR-616-associated suppression of antigen-specific IgG responses following KLH immunization in mice and monkeys. Additionally, no cases of infection were reported in monkey studies after 9 months of KZR-616 treatment (data not shown). Coupled with the reduction of activated B cells and short- and long-lived plasma cells we observed, our data suggests that effects specific to pathogenic antibody producing cells represent a key function by which KZR-616 impacts disease progression without inducing immunosuppression. The mechanisms for this effect remain to be fully elucidated but may be a result of the recovery of immunoproteasome function between the weekly dose administrations.

We monitored whole transcriptome changes in mouse blood, spleen, and kidney, as well as compared changes between diseased and pre-disease animals to elucidate common, disease-related pathways that are sensitive to KZR-616. Consistent with our *in vitro* human T and B cell differentiation data and flow cytometric analysis of lymphoid tissues from diseased mice, we found that KZR-616 modulates multiple innate and adaptive immune signaling pathways. These included broad changes in T cell function, as well as Th differentiation. Strikingly, the ASC module was the most significantly downregulated module in the spleen. Amongst the plasma cell-related genes inhibited by KZR-616 were Blimp (Prdm1), Il21, Xbp1, and Irf4, which all play a role in the differentiation of this cell type, mirroring the effects we saw *in vitro* on plasmablast formation (**Table S2**) (47–49). Additionally, we observed reduced mRNA levels of BAFF (Tnfsf13b), IL6, APRIL (Tnfsf13), BCMA (Tnfrsf17), TACI (Tnfrs13B), and Cxcr3, genes that support the survival, maturation, and differentiation of plasma cells (**Table S2**) (50). It was previously reported that ONX 0914 reduced plasma cell numbers and levels of these proteins in a rat allograft model (36, 46, 51). Therefore, we surmise that these plasma cell effects represent a common pathway for the anti-inflammatory activity of KZR-616 across immune-mediated diseases. Additionally, changes in gene modules for plasmacytoid dendritic cells (pDCs) and interferon signaling observed in blood and tissues are consistent with previous findings of inhibition of IFN-α release by stimulated human pDC and mouse splenocytes following ONX 0914 treatment (13). Thus, KZR-616 represents an agent that impacts both adaptive immune responses and Type I IFN pathways, which are each targets of extensive investigation in the setting of SLE (52, 53).

Global gene expression profiling enabled us to identify multiple additional KZR-616-sensitive pathways known to play a role in SLE pathogenesis that were not revealed by analysis of cytokine levels or immune effector cell phenotyping. Within the spleen this included CD28 and ICOS-ICOSL signaling in T helper pathways, which are both dysregulated in SLE and are the targets of experimental therapeutics (54, 55). Intriguingly, KZR-616 treatment was associated with upregulation of multiple erythrocyte modules. Like the NZB/W F1 mice, anemia is a common finding in SLE patients, suggesting that KZR-616 may impact multiple serologic markers of disease beyond autoantibody levels (56). In addition, multiple pathways associated with oxidative phosphorylation, cell cycle, and cellular stress were upregulated following KZR-616 treatment, which may relate to the striking effects of KZR-616 on morphologic changes within the kidney of diseased animals. Many of the immunologic pathways modulated in the spleen of NZB/W F1 mice treated with KZR-616 were also found to be aberrantly expressed in other tissues from SLE patients, including the skin and synovium. Taken together, our data suggest that the immunoproteasome regulates the function of both innate and acquired immune responses, as well as cellular metabolism pathways, *via* regulation of multiple survival and differentiation factors.

Within the target tissue of disease, we found that therapeutic benefit induced by KZR-616 was associated with reduced expression of multiple gene modules that are aberrantly expressed in the kidneys of LN patients (31). These gene changes were not restricted to innate and adaptive immune modules, but also involved tissue repair and remodeling. Previous studies in NZB/W F1 mice have shown that as glomerular inflammation progresses, downstream peritubular blood flow is associated with oxidative and metabolic stress, mitochondrial dysfunction, and tubular damage (57). We found that KZR-616 treatment led to the upregulation of several mitochondrial and metabolic modules, suggesting that the prevention of kidney damage by KZR-616 may be mediated in part by reversal of these metabolic changes. In total, KZR-616 reduced the majority of the genes in glomerular and tubulointerstitial modules previously identified to be upregulated in LN patients (31).

To set expectations for biomarker analysis from our clinical studies, we sought to identify common gene and gene module changes in mouse whole blood, spleen and kidneys. Interestingly, we found that only a small fraction of genes differentially expressed across all 3 tissue types, or even between blood and kidneys. However, the modules that are shared across tissues are representative of the major pathways impacted by KZR-616 and, importantly, those altered in mice as they develop disease. The relatively low degree of overlap between tissues is not entirely unexpected given baseline differences in the gene expression profiles of these tissues, but it does help to clarify how often and for which genes and gene modules we should expect blood to be a useful surrogate for spleen and kidney tissue in human transcriptomic studies.

Finally, we found that upon subcutaneous administration to healthy volunteers, KZR-616 induced a similar profile of selective and potent inhibition of the immunoproteasome as seen *in vitro* and in mice, marking the first time that selective inhibition of this target has been achieved in humans. As observed in our preclinical studies, this profile of inhibition resulted in a broad anti-cytokine response when assessed *via ex vivo* stimulation of immune effector cells.

In conclusion, KZR-616 is a novel and selective inhibitor of the immunoproteasome that potently blocks inflammatory cytokine production *in vitro* and disease progression in mouse models of SLE *via* modulation of multiple pathways associated with T and B cell signaling, innate and adaptive immunity, mitochondrial dysfunction, and glomerular and tubulointerstitial injury. The data presented here suggest that KZR-616 has the potential to be therapeutically active across multiple autoimmune indications while avoiding immune suppression, and can be administered with other active agents, such as MMF. These data support the ongoing study of KZR-616 in patients with LN.

## Data availability statement

The original contributions presented in the study are included in the article/**Supplementary Material**. Further inquiries can be directed to the corresponding authors.

## Ethics statement

The studies involving human participants were reviewed and approved by The Alfred Hospital Ethics Committee. The patients/participants provided their written informed consent to participate in this study. The animal study was reviewed and approved by Kezar Life Sciences IACUC.

## Author contributions

TM, RF, EL, BT, and BM designed and performed the experiments. RF performed *in vitro* human T and B cell differentiation studies. EL performed ProCISE assays for human PBMCs. BM performed human normal and SLE PBMC stimulation assays. TM performed mouse lupus studies. BT and TM performed the bioinformatic analyses. DB oversaw the phase I clinical trial. JA directed and analyzed LLVY and ProCISE assays of clinical trial samples, performed by CRO (Eurofins Bioanalytical Services, Oxford, UK). RF designed and directed *ex vivo* human blood stimulation studies, which were developed and optimized by EL and performed by CRO (Nucleus Network, Melbourne, Victoria, Australia). DM and HJ conceived the KZR-616 chemical series and synthesis. CK oversaw all research studies. RF, BT, CK, and TM wrote the manuscript. All authors contributed to the article and approved the submitted version.
