# Supplementary material for: Zetomipzomib (KZR-616) attenuates lupus in mice via modulation of innate and adaptive immune responses
Source: Front Immunol. 2023 Mar 10;14:1043680. doi: 10.3389/fimmu.2023.1043680 (PMC10036830; doi:10.3389/fimmu.2023.1043680)
Supplement: Supplementary file 1 [file DataSheet_1.pdf]

Supplemental methods

Evaluation of the consequences of KZR-616 treatment on T cell dependent antibody responses (TDAR).

Male CD1 (ICR) mice were treated with 10 mg/kg KZR-616 SC once weekly for 4 consecutive weeks. On Days 14 and 21, mice were immunized intraperitoneally with 100 µg keyhole limpet hemocyanin [KLH; Thermo Scientific (Pierce)]. Serum samples were collected predose and on Days 21 and 29. Anti- KLH antibodies and concentrations were analyzed by a commercially available ELISA（Life Diagnostics Inc.). The study was performed at Charles River Laboratories.

Male cynomolgus monkeys were administered KZR-616 SC once weekly starting on Day 0 for 39 consecutive weeks. Monkeys were then immunized with KLH on Days 180 (primary immunization) and 208 (secondary immunization) and blood was collected for analysis of anti-KLH antibodies (IgM and IgG) on Days 180 (pre-KLH), 187, 190, 194, 201, and 208 (7, 10, 14, 21, and 28 days post-primary and pre-secondary KLH immunization, respectively), and on Days 215, 218, 222, 229, and 236 (7, 10, 14, 21, and 28 days post-secondary KLH immunization, respectively). Anti-KLH IgM and IgG antibodies were determined by ELISA. In brief, he KLH coated microtiter plate containing the diluted samples and controls (100 µL/well) were plated within 4 hours of preparation and incubated for 25 to 35 minutes at ambient temperature. Subsequently, the plates were washed 4 times to remove unbound sample/control using 300 µL of 1X phosphate buffered saline tween 80 (PBS-T) per wash. Goat anti-monkey IgM-horseradish peroxidase (HRP) or goat anti-monkey IgG-HRP were added at 100 µL per

well and the plates were incubated at ambient temperature for 25 to 35 minutes. After aspiration of the solution from the plate, the plates were washed 6 times with 300 µL per well of 1X PBS-T per wash and were developed by addition of 100 µL of the substrate 3, 3′5, 5′ tetramethylbenzidine (TMB) for 10 to 14 minutes for IgM or 7 to 13 minutes for IgG. The substrate and HRP reaction were inactivated (stopped) by the addition of 100 µL of 2N sulfuric acid (H2SO4), and the mean absorbance values were quantified at 450 nm. Anti-KLH IgM and IgG antibodies concentrations are shown for n = 7 monkeys per group. The study was performed at Charles River Laboratories.

Detection of antibody-secreting cells by ELISPOT

Anti-dsDNA secreting cells were detected as previously described (Becker 2010) Spleen cell suspensions were incubated as serial dilutions starting with $5x10^5$ cells/well overnight at 37C. After incubation, plates were washed and incubated with alkaline phosphatase-conjugated goat antibody to mouse IgG (Jackson ImmunoResearch Labs, West Grove, PA) for 1 hr at RT and detected with Vector Blue Alkaline Phosphatase Substrate Kit III (Burlingame, CA). Anti-IgG secreting cells were assessed with serial dilutions starting at $1x10^5$ cells/well and detected using a commercially available ELISPOT kit from Immunospot (Cellular technology Limited (CTL), Cleveland, OH). The developed spots were measured by ImmunoSpot 5.0 (Cellular Technology Limited).

Supplemental Figures

Supplemental Figure 1: Structure of KZR-616

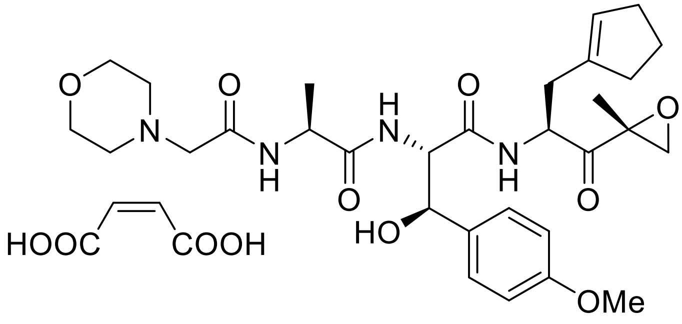

KZR-616 Maleate

Supplemental Figure 2a.

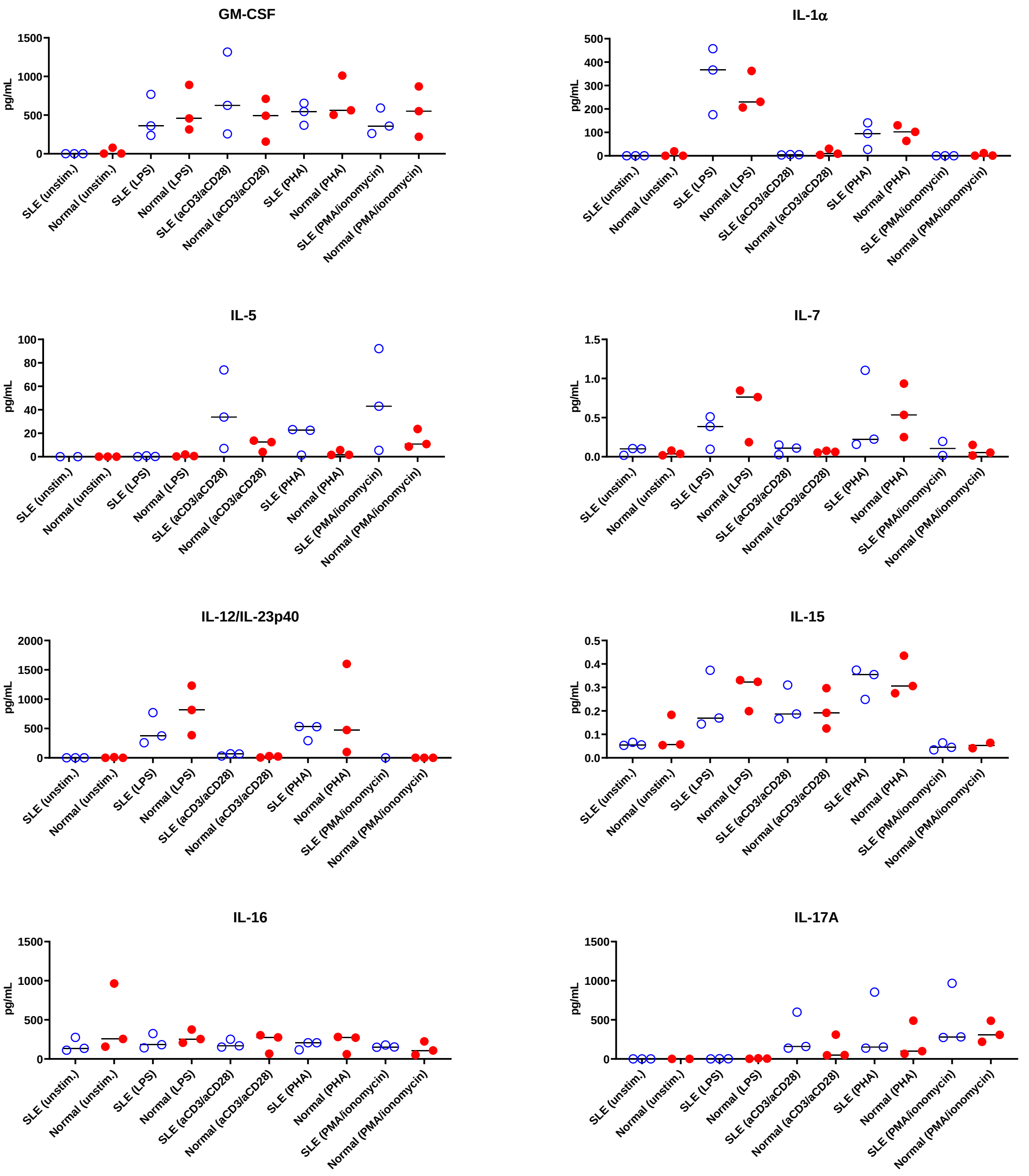

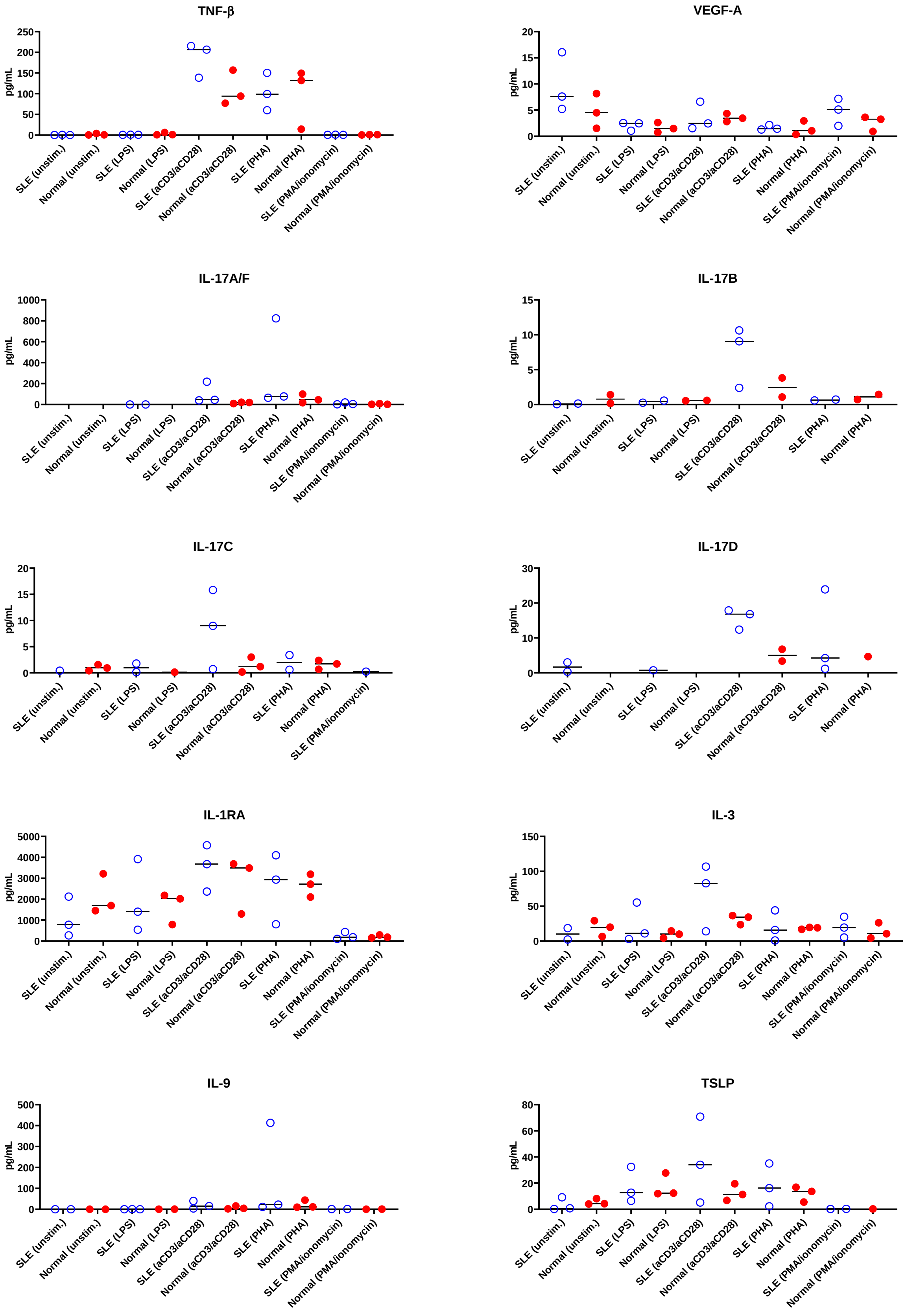
TNF-β
VEGF-A
IL-17A/F
IL-17B
IL-17C
IL-17D
IL-1RA
IL-3
IL-9
TSLP
pg/mL
SLE (unstim.)
Normal (unstim.)
SLE (LPS)
Normal (LPS)
SLE (aCD3/aCD28)
Normal (aCD3/aCD28)
SLE (PHA)
Normal (PHA)
SLE (PMA/ionomycin)
Normal (PMA/ionomycin)

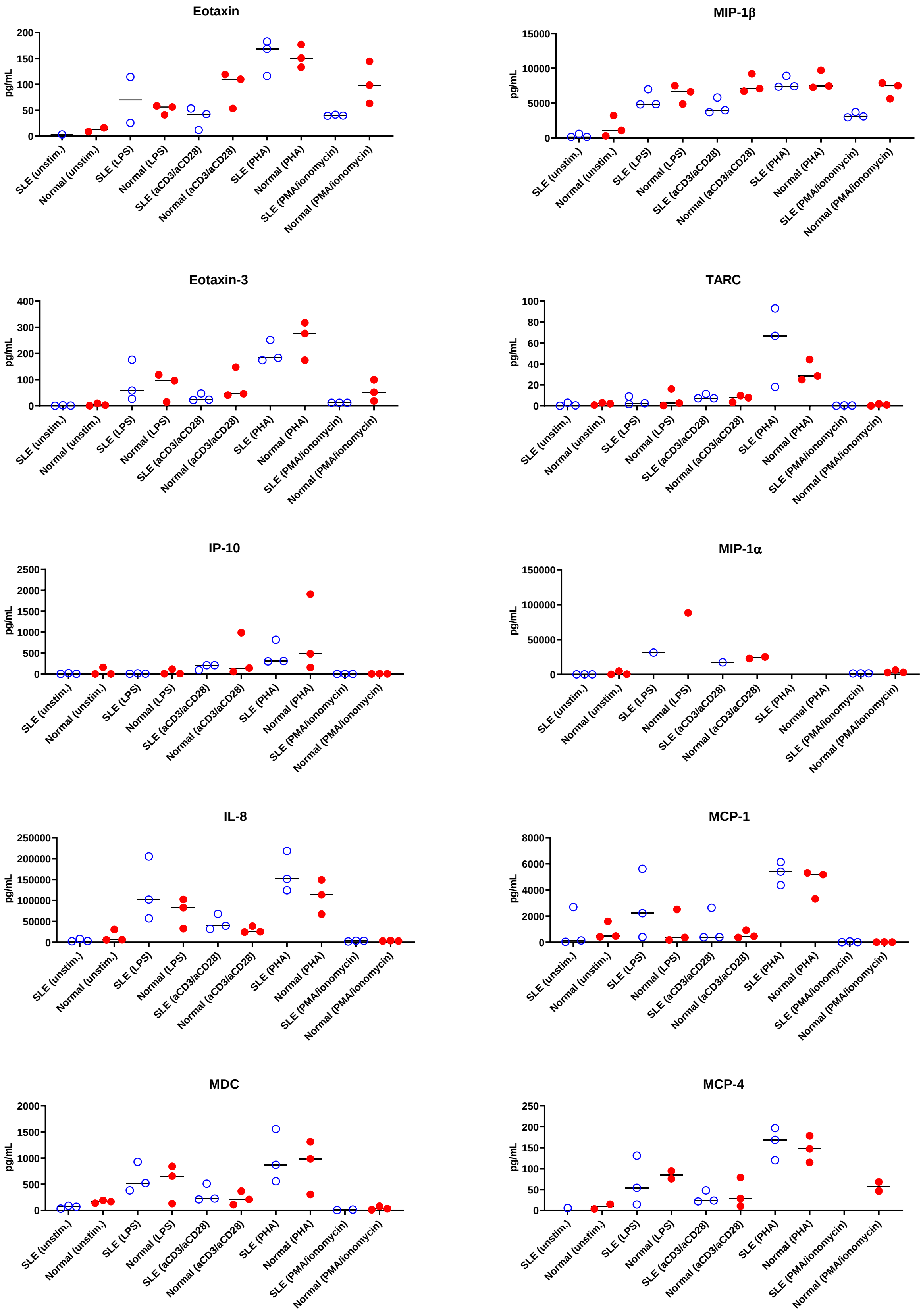

Eotaxin
MIP-1β
Eotaxin-3
TARC
IP-10
MIP-1α
IL-8
MCP-1
MDC
MCP-4
pg/mL
SLE (unstim.)
Normal (unstim.)
SLE (LPS)
Normal (LPS)
SLE (aCD3/aCD28)
Normal (aCD3/aCD28)
SLE (PHA)
Normal (PHA)
SLE (PMA/ionomycin)
Normal (PMA/ionomycin)
0
50
100
150
200
250
300
400
500
1000
1500
2000
2500
5000
10000
15000
20
40
60
80
50000
100000
150000
200000
250000
2000
4000
6000
8000

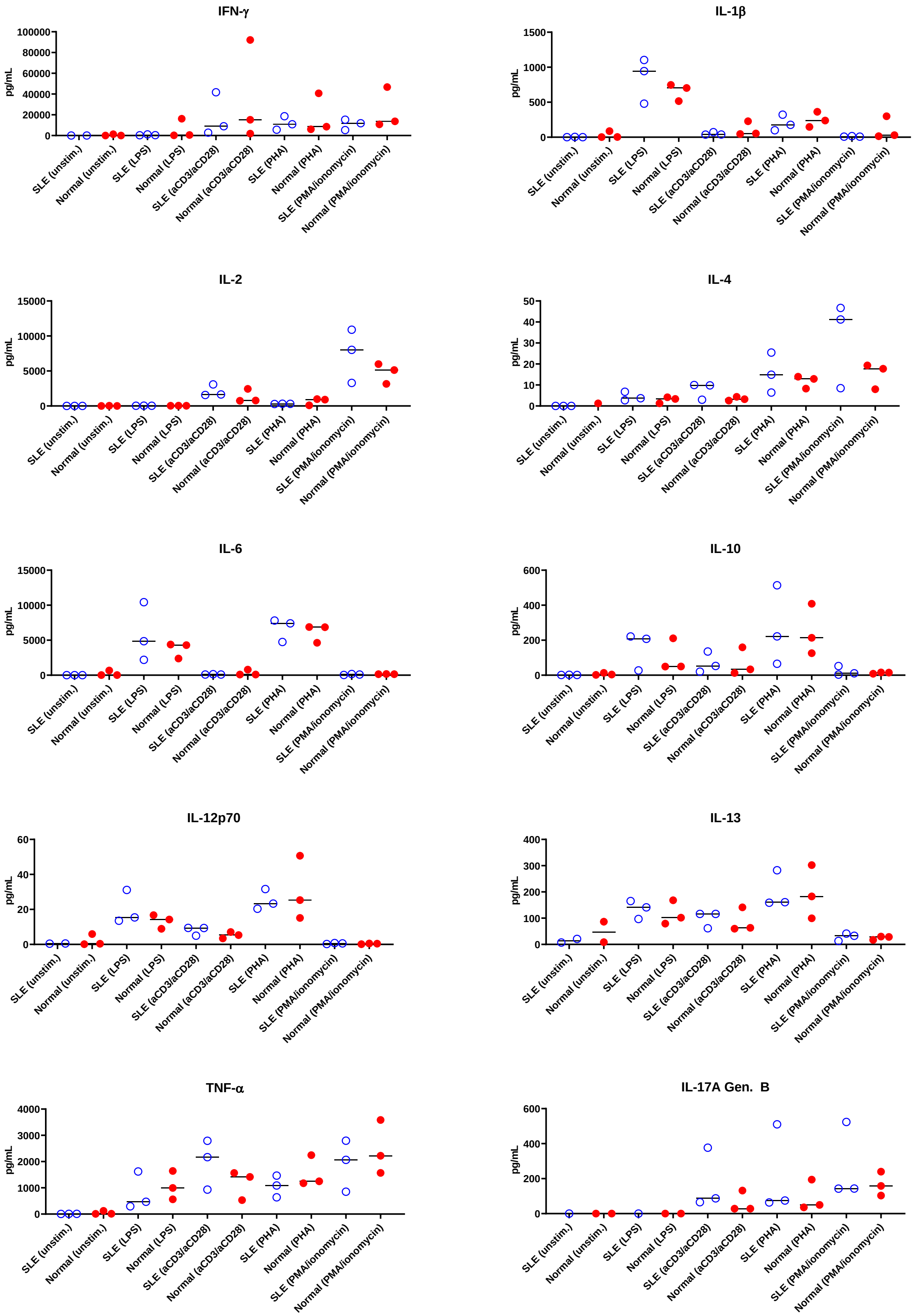
IFN-γ
IL-1β
IL-2
IL-4
IL-6
IL-10
IL-12p70
IL-13
TNF-α
IL-17A Gen. B
pg/mL
SLE (unstim.)
Normal (unstim.)
SLE (LPS)
Normal (LPS)
SLE (aCD3/aCD28)
Normal (aCD3/aCD28)
SLE (PHA)
Normal (PHA)
SLE (PMA/ionomycin)
Normal (PMA/ionomycin)

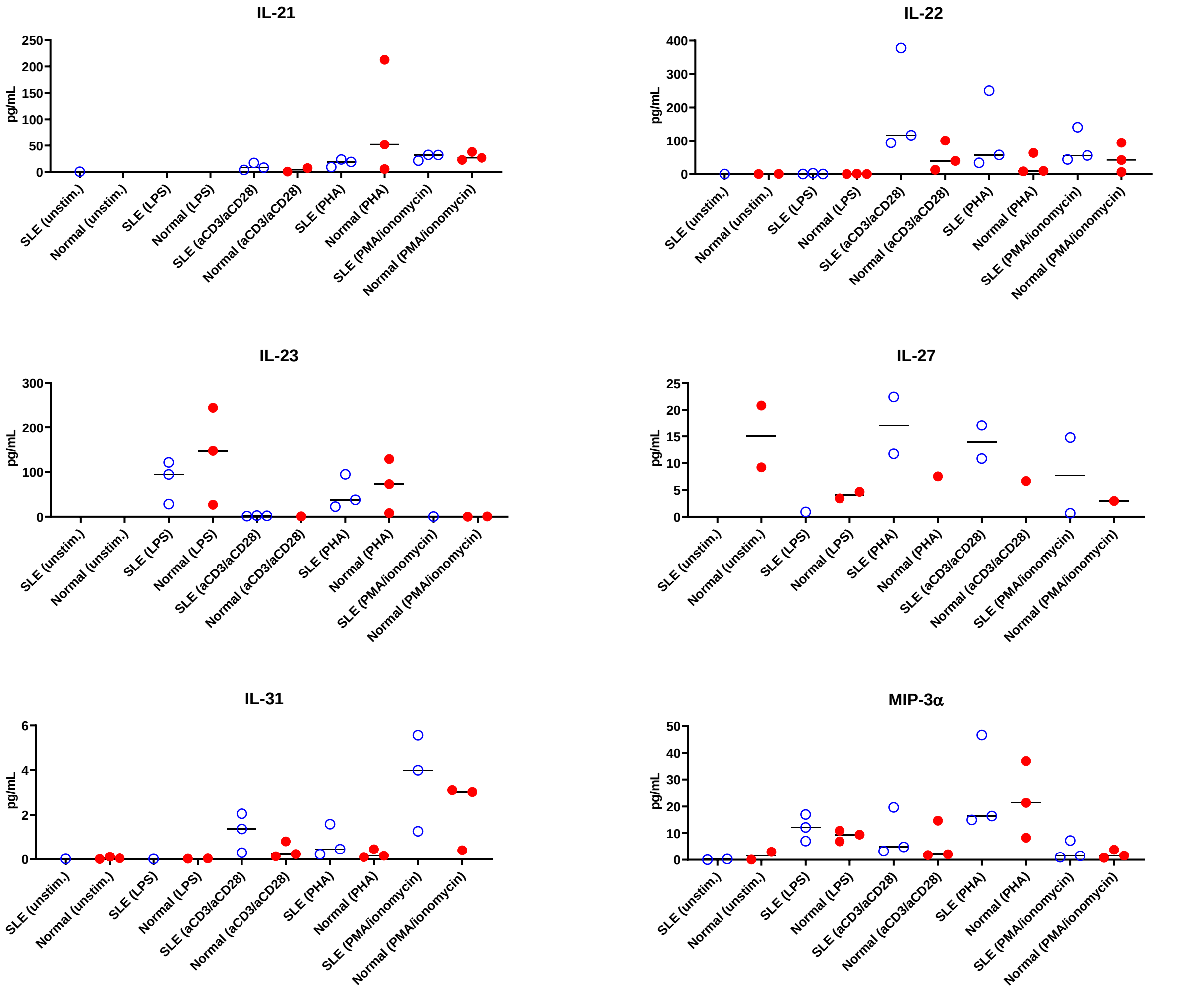

Supplemental Figure 2a. Baseline cytokines in SLE and HV PBMC with or without stimulation (SLE in blue, HV in red). PBMCs from 3 HV or 3 SLE patients were unstimulated or stimulated with LPS, anti-CD3 and anti-CD28, PHA or PMA plus ionomycin. PBMC Supernatants were collected and analyzed for cytokine release by multiplexed electrochemiluminescent immunoassay detection (44-plex cytokine/chemokine panel).

Supplemental Figure 2b.

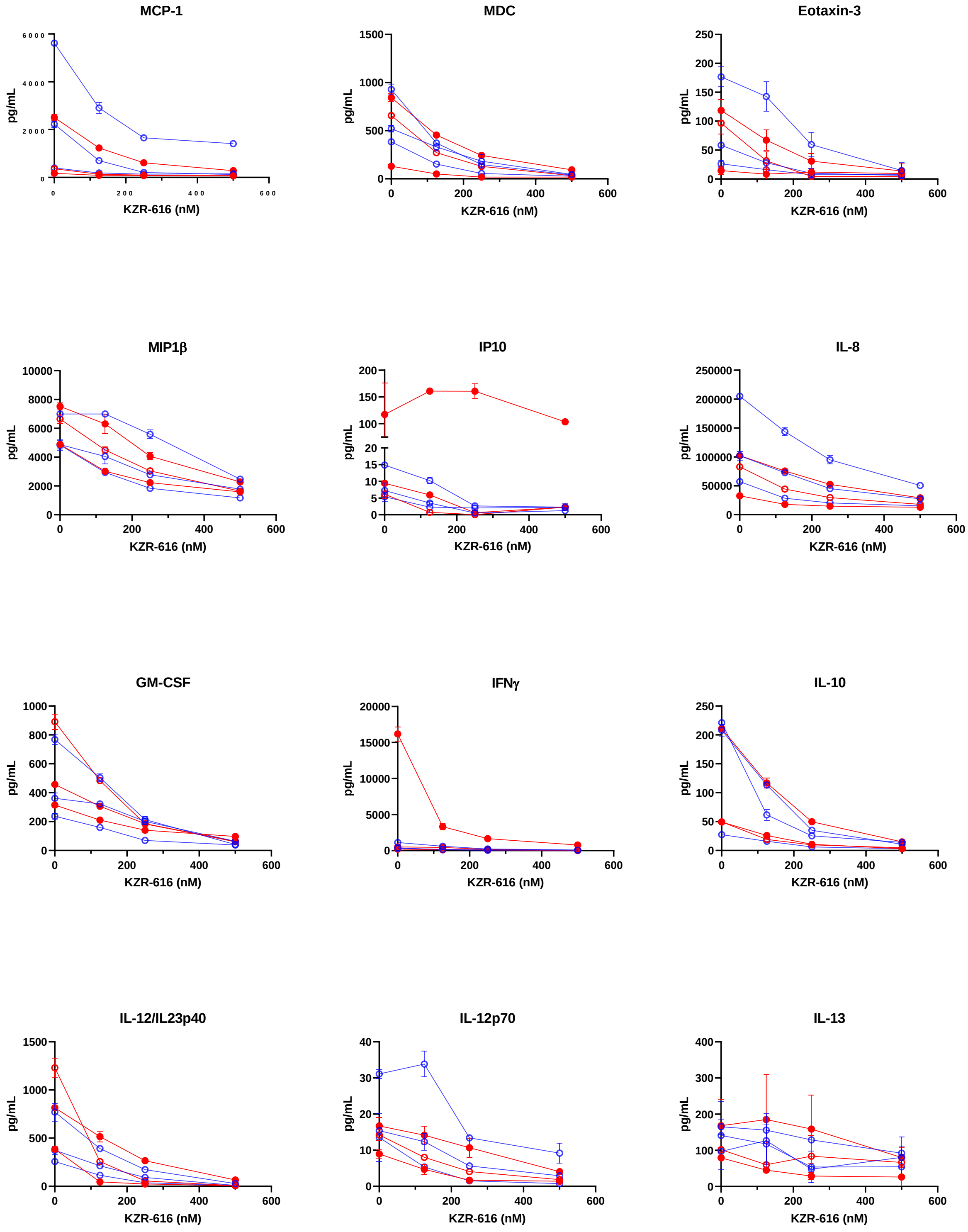

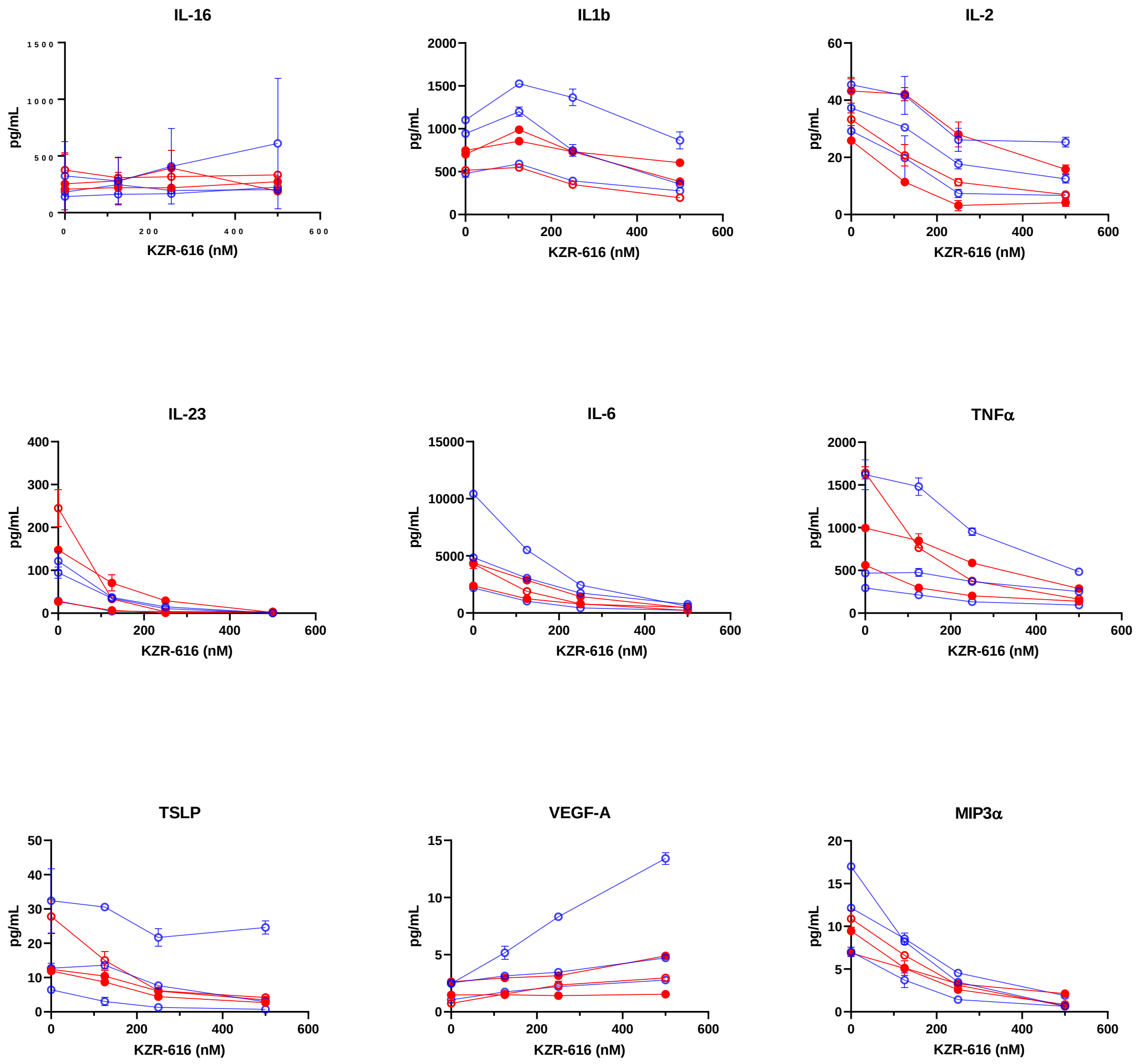

Supplemental Figure 2b. KZR-616 effect on in SLE and HV PBMC with LPS stimulation (SLE in blue, HV in red). PBMCs from 3 HV or 3 SLE patients were pre-treated with media control or with KZR-616 (125, 250 and 500nM), washed and stimulated with LPS, PBMC Supernatants were collected and analyzed for cytokine release by multiplexed electrochemiluminescent immunoassay detection (44-plex cytokine/chemokine panel). Cytokines detected with >5-fold change over unstimulated control are shown.

Supplemental Figure 2c.

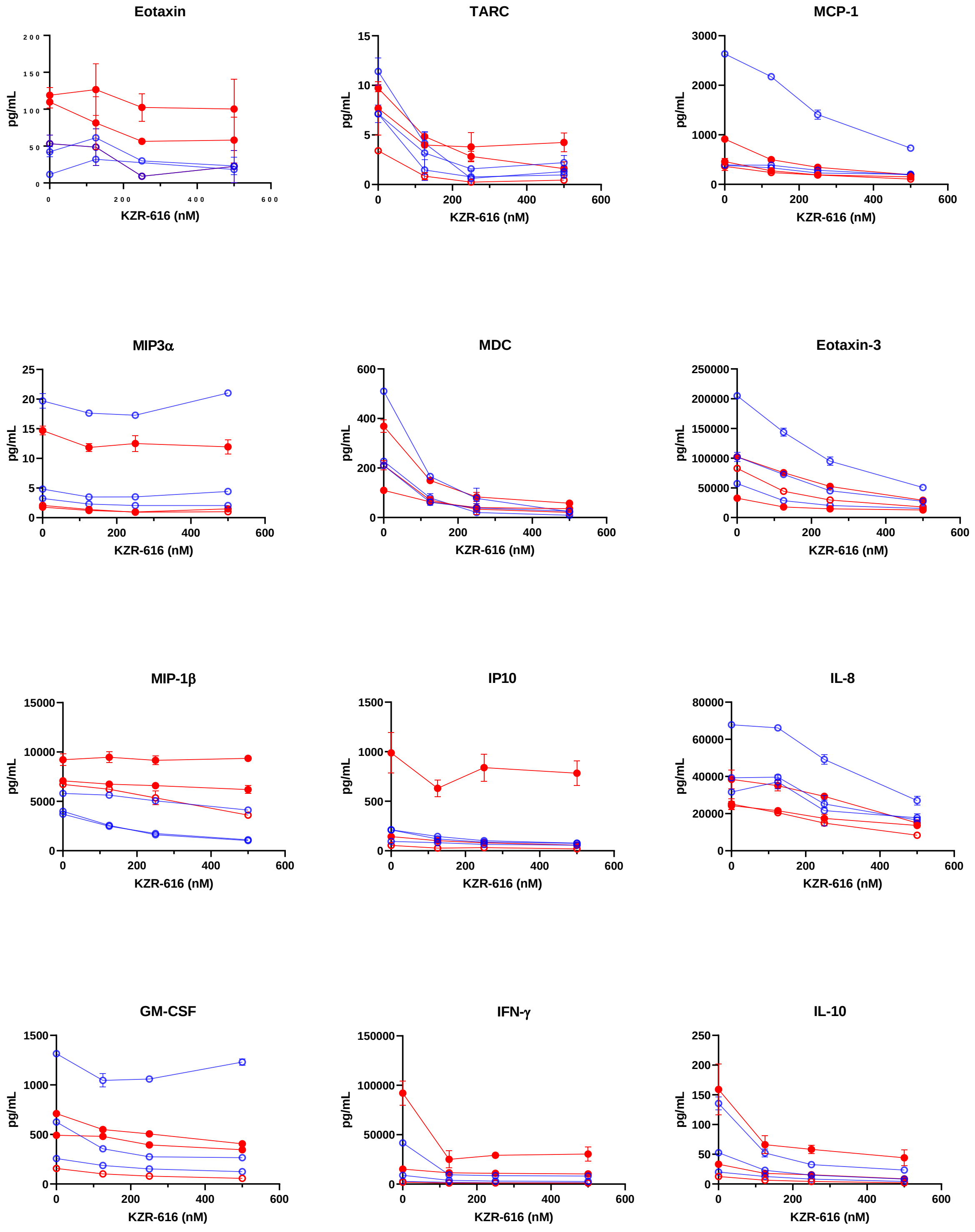

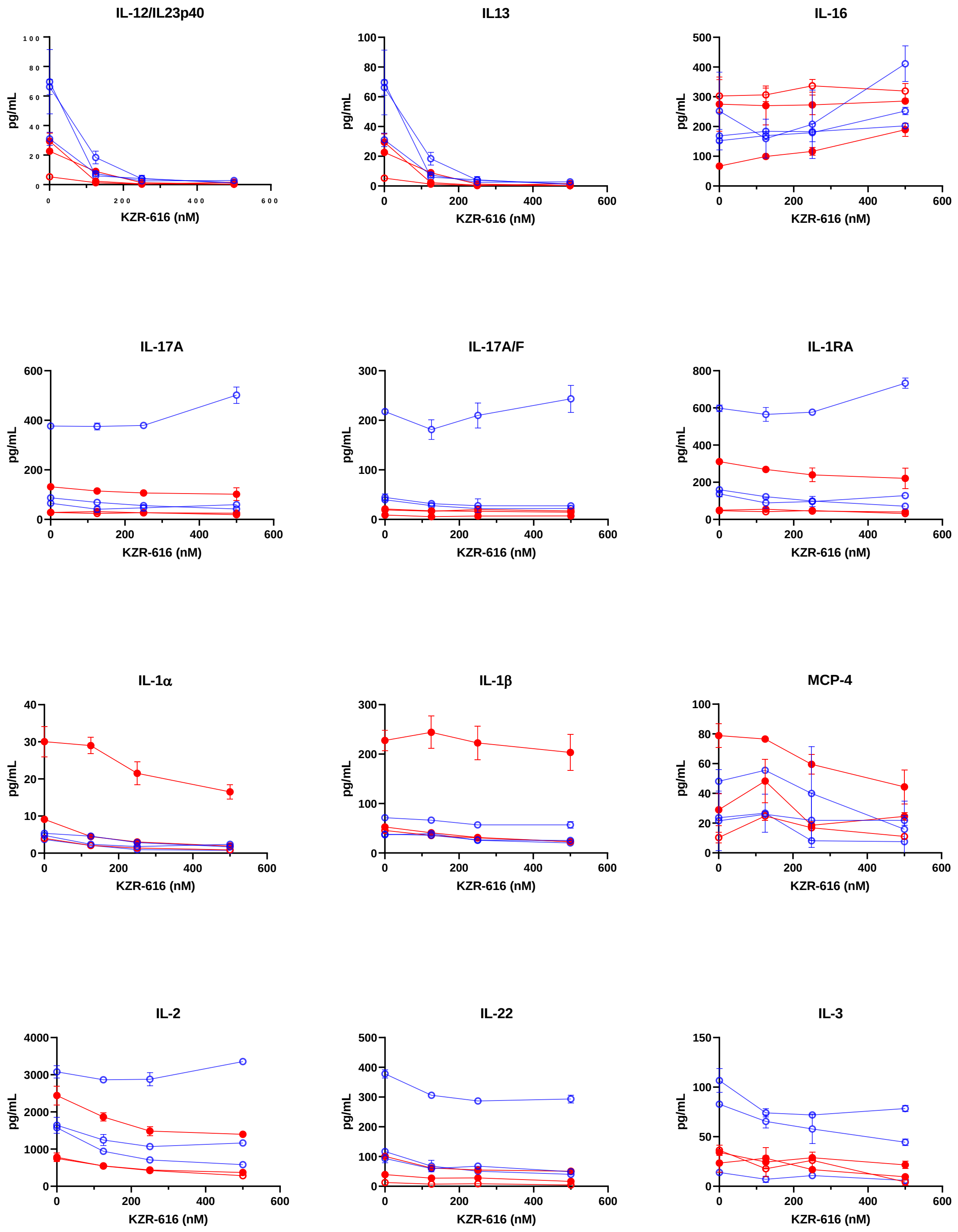
IL-12/IL23p40
pg/mL
KZR-616 (nM)
IL13
pg/mL
KZR-616 (nM)
IL-16
pg/mL
KZR-616 (nM)
IL-17A
pg/mL
KZR-616 (nM)
IL-17A/F
pg/mL
KZR-616 (nM)
IL-1RA
pg/mL
KZR-616 (nM)
IL-1α
pg/mL
KZR-616 (nM)
IL-1β
pg/mL
KZR-616 (nM)
MCP-4
pg/mL
KZR-616 (nM)
IL-2
pg/mL
KZR-616 (nM)
IL-22
pg/mL
KZR-616 (nM)
IL-3
pg/mL
KZR-616 (nM)

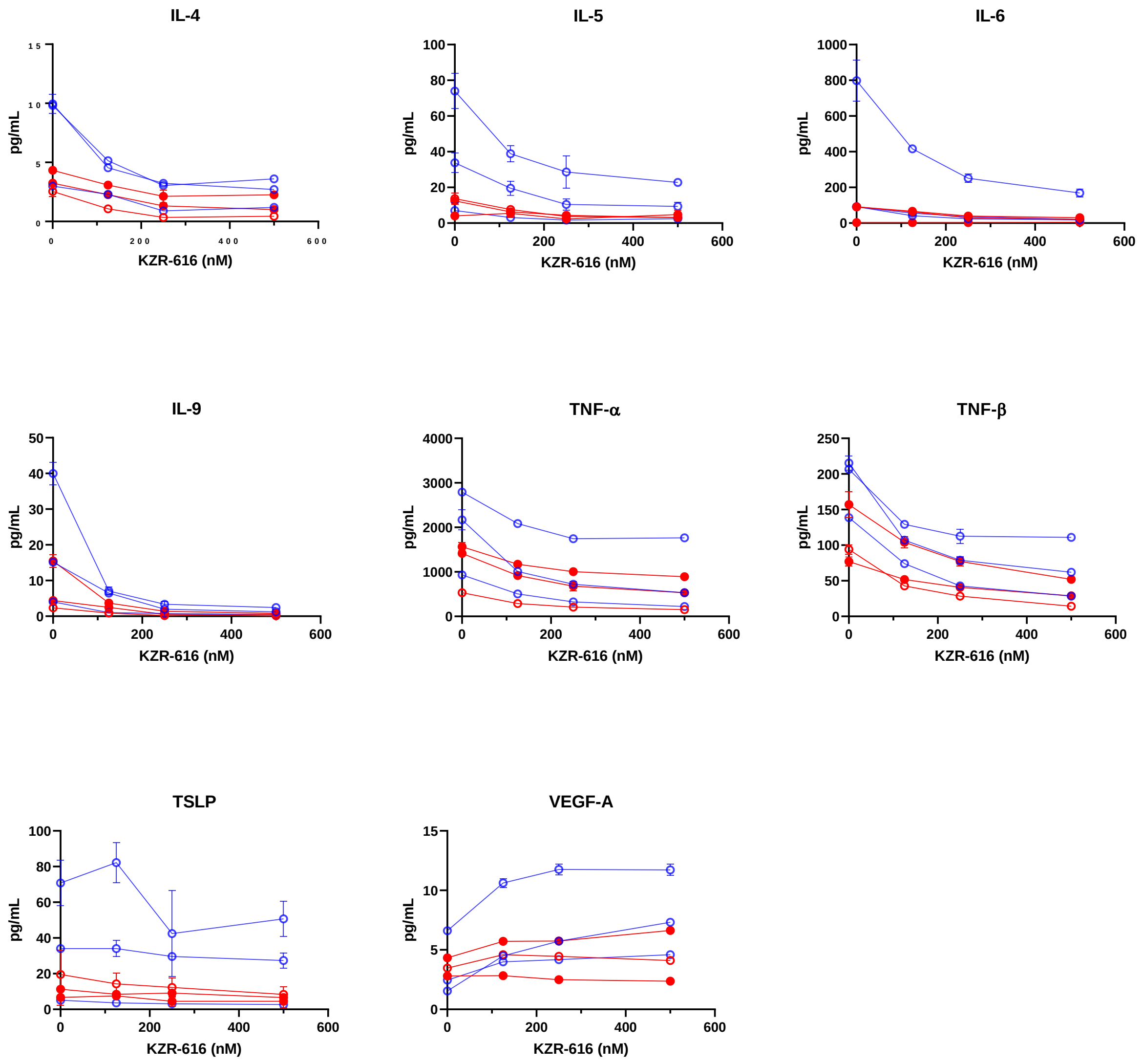

Supplemental Figure 2c. KZR-616 effect on in SLE and HV PBMC with anti-CD3 and anti-CD28 stimulation (SLE in blue, HV in red). PBMCs from 3 HV or 3 SLE patients were pre-treated with media control or with KZR-616 (125, 250 and 500nM), washed and stimulated with anti-CD3 and anti-CD28, PBMC Supernatants were collected and analyzed for cytokine release by multiplexed electrochemiluminescent immunoassay detection (44-plex cytokine/chemokine panel). Cytokines detected with >5-fold change over unstimulated control are shown.

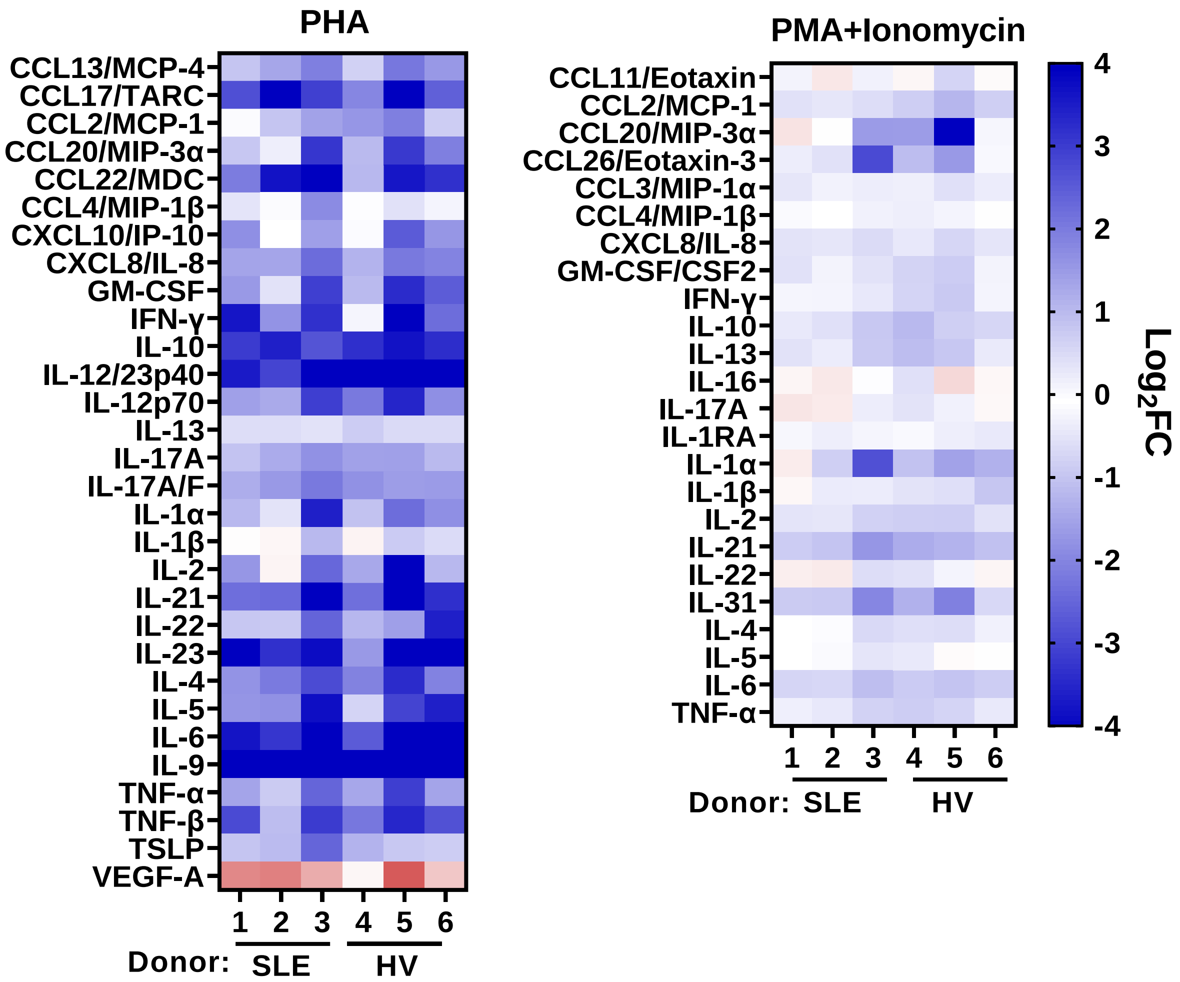

Supplemental Figure 2d. KZR-616 blocks cytokine production in human PBMCs (PHA; PMA and ionomycin) PBMCs from 3 HV or 3 SLE patients were treated with 500 nM KZR-616 for 1 hour as described in Figure 1, then stimulated with PHA (24 h) or PMA and ionomycin (4 hours). Cytokines detected with >5-fold change over unstimulated control are shown and presented as log2 fold-change relative to stimulated DMSO control.

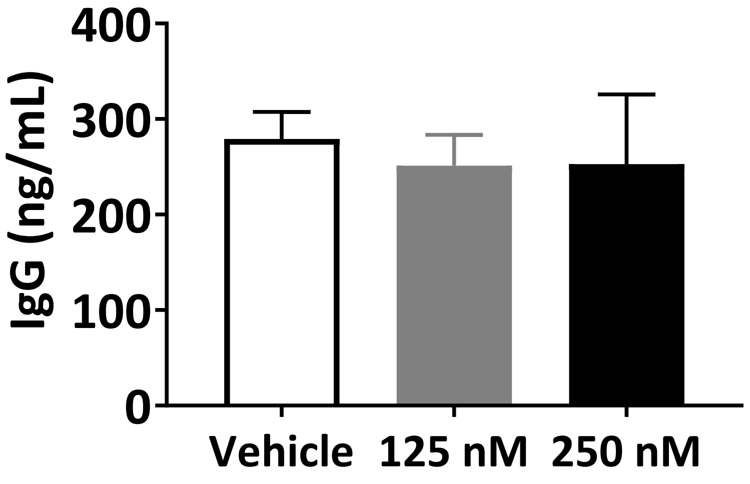

Supplemental Figure 3. KZR-616 does not inhibit human B plasmablast IgG secretion. Human peripheral blood $CD19^+$ B cells were pre-differentiated into IgG-secreting plasmablasts in vitro with anti-CD40 and anti-IgM antibodies in the presence of IL-21 for 6 days. Plasmablasts were treated with vehicle or KZR-616 as a 1 hour pulse and incubated for 24 hours. Secretion of IgG into culture media was measured by MSD immunoassay. Representative of N=4 independent experiments are shown.

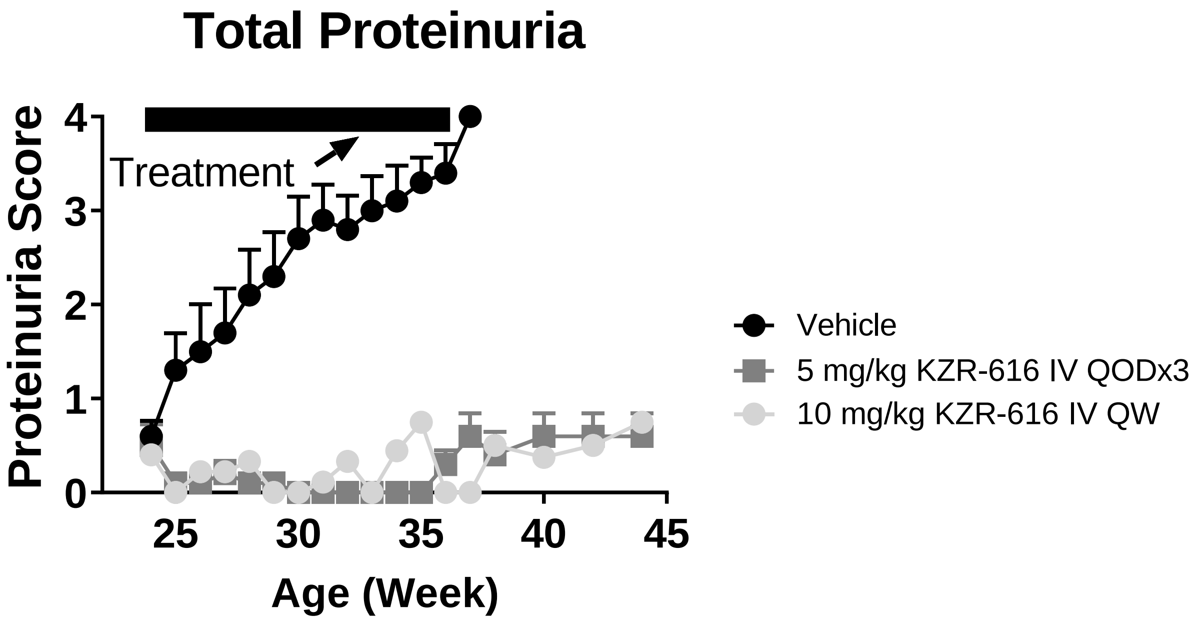

Supplemental Figure 4. Once weekly administration of KZR-616 reduces proteinuria similarly to thrice weekly administration in lupus prone mice. (A) NZB/W F1 mice (proteinuria grade 1 at study start) were treated QODx3 with vehicle (IV), QODX3 with KZR-616 (5 mg/kg) or QW with KZR-616 (10 mg/kg) for 13 weeks followed by an 8-week non-dosing period for KZR-616 (n=10/group). Data are presented as mean proteinuria scores (±SEM).

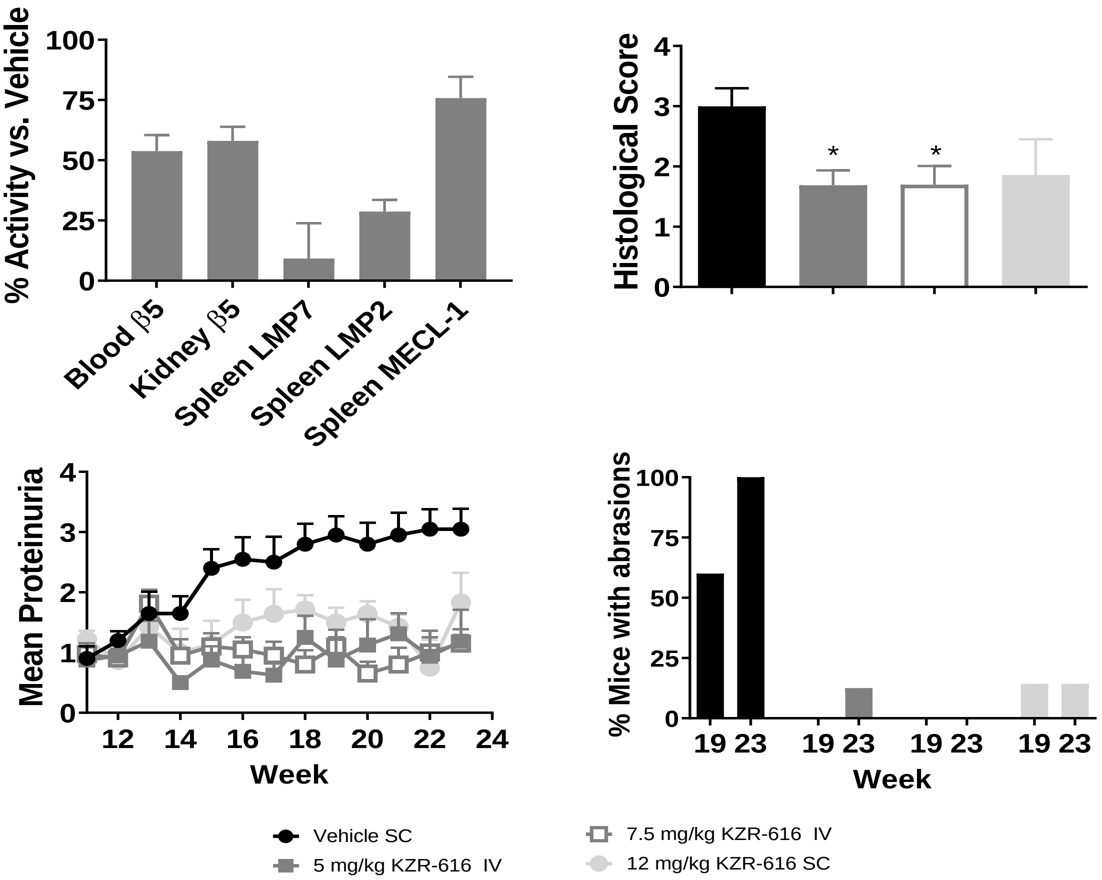

Supplemental Figure 5. KZR-616 ameliorates nephritis progression in MRL/lpr lupus prone mice. (A) MLR/lpr were intravenously administered 5 mg/kg of KZR-616. Kidney and splenocyte (erythrocyte-depleted) samples were taken 1 h after dosing, and the activity of LMP7, LMP2, MECL-1 (splenocytes), and β5 (kidney) was measured by ProCISE. Data were normalized to the average activity of vehicle-treated animals and are presented as the average relative activity ± SD (n = 3). **(B)** MRL/lpr mice (proteinuria grade 1) were treated with vehicle (SC) QODx3 or KZR-616 (5 mg/kg IV QODx3, 7.5 mg/kg IV QW or 12 mg/kg SC QW) for 11 weeks (n=10/group). **(C)** Histological changes were scored from 0-4 for glomerular nephritis which included tubular changes and lymphoid infiltrates following 13 weeks of treatment. **(D)** Percent of mice with skin abrasions on weeks 19 and 23. Data are presented as mean scores (±SEM). * P<0.01 and **P<0.002 by using one-way ANOVA with Dunnett's correction (Figure B).

A

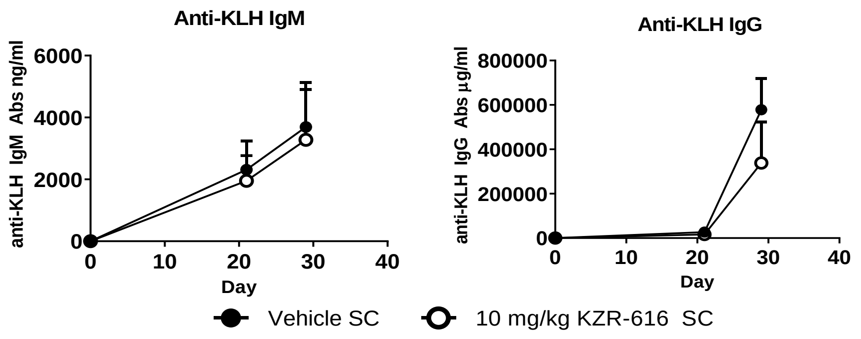

B

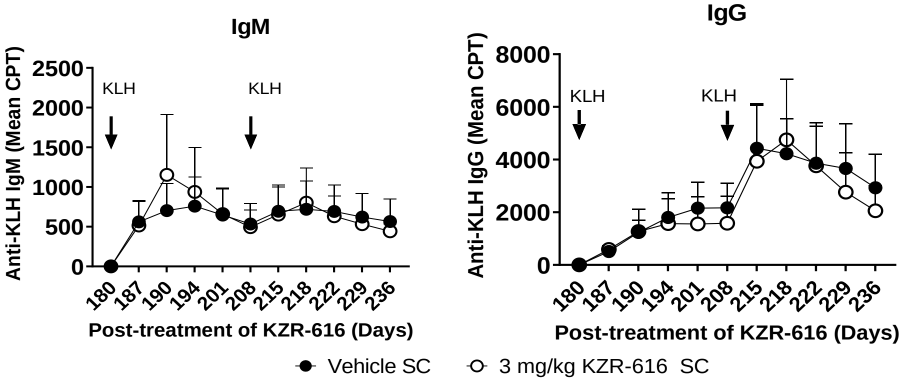

Supplemental Figure 6. Effects of KZR-616 on T cell dependent antibody responses in mice and monkeys. **(A)** BALB/c mice were immunized with keyhole limpet hemocyanin (KLH) on Days 14 and 21. KZR-616 (10 mg/kg SC) was administered once a week for four consecutive weeks. Mean (± SEM) serum anti-KLH IgM and IgG antibody levels are shown (n = 10/group). **(B)** Male cynomolgus monkeys were administered 3 mg/kg KZR-616 SC once weekly starting on Day 0 for 39 consecutive weeks. Animals were immunized with KLH on Days 180 (primary immunization) and 208 (secondary immunization) and blood was collected for analysis of anti-KLH antibodies (IgM and IgG) on Days 180 (pre-KLH), 187, 190, 194, 201, and 208 (7, 10, 14, 21, and 28 days post-primary and pre-secondary KLH immunization, respectively), and on Days 215, 218, 222, 229, and 236 (7, 10, 14, 21, and 28 days post-secondary KLH immunization, respectively). Mean (± SEM) serum anti-KLH IgM and IgG antibodies levels are shown (n = 7/group).

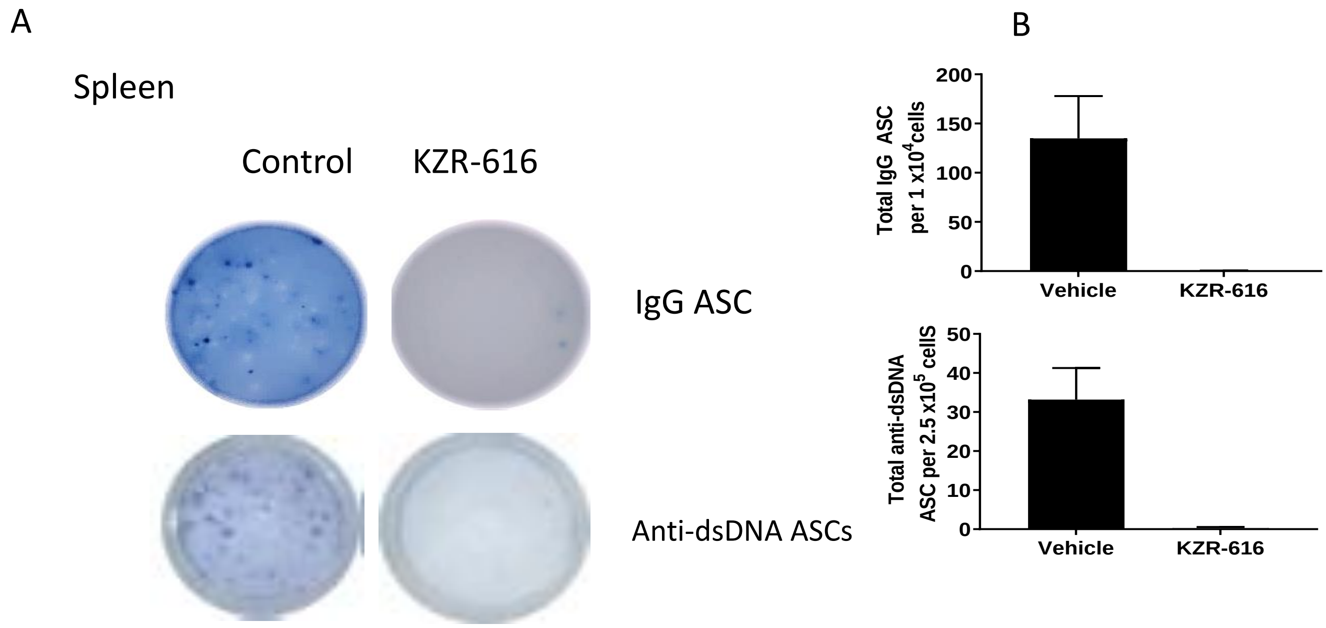

Supplemental Figure 7. KZR-616 Treatment Decreases ASCs in spleen. NZB/W mice (proteinuria grade 1) were treated with vehicle or 5 mg/kg IV KZR-616 QODx3 for 13 weeks. Spleen cells were serially titrated into a 96-well plate and stained as previously described. **(A)** Representative wells of ELISPOT results for IgG and anti-dsDNA (5 x 10^5 cells/well). **(B)** Total IgG (per 1x10^4 cells) and anti-dsDNA ASC cells (per 2.5 x10^5) in spleen from NZB/W mice treated with vehicle or KZR-616. Data are presented as mean scores (±SEM).

Supplemental Figure 8. Full list of ASC gene transcripts in spleen following KZR-616 treatment in NZB/W F1 Mice

Heatmap visualization of select ASC-specific transcripts that were differentially-expressed (adjusted P-value < 0.01 and fold-change ≥ 2) in spleens of vehicle- and KZR-616-treated animals. Each column represents 1 animal. Genes marked in blue were reduced after KRZ-616 treatment compared to vehicle-treated animals (fold change ≥ 2, P < 0.01).

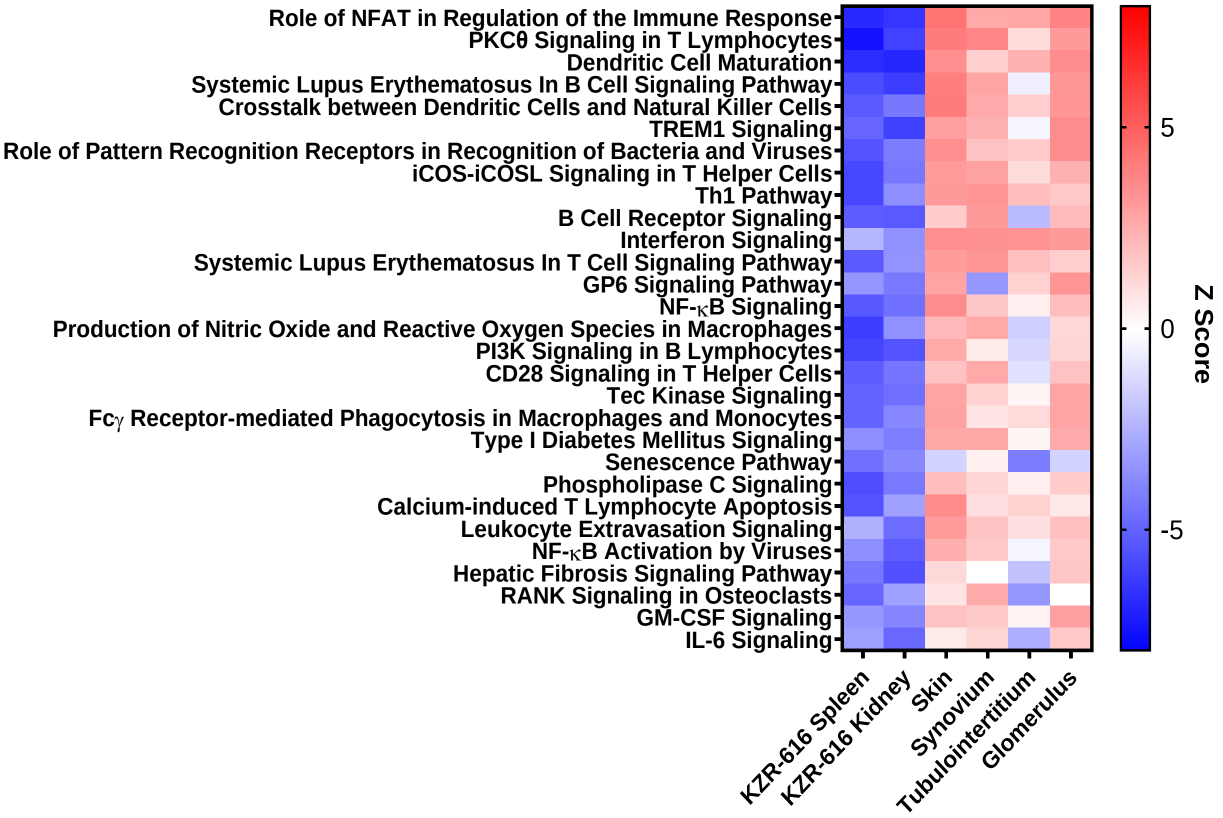

Supplemental Figure 9. KZR-616 Treatment decreases lupus gene signature pathways in NZB/W mice that are increased in active human SLE. RNA sequencing analysis was performed on spleen and kidney (n=5-6/group) derived from the experiment described in Figure 5. IPA match analysis was performed between the differentially-expressed transcripts from KZR-616-treated NZB/W F1 mice and the profiles from human SLE microarray tissue datasets. The top 30 most significantly decreased and increased pathways by Z score are listed. scores greater or <2 are considered significant.

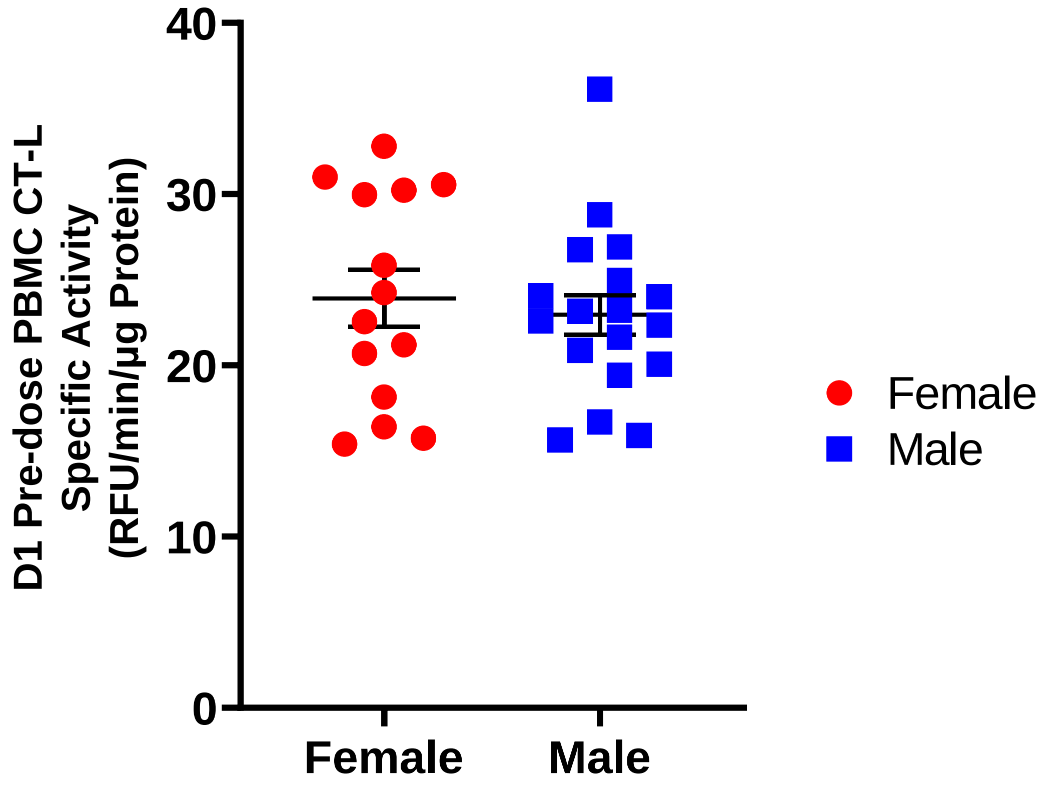

Supplemental Figure 10. Baseline immunoproteasome activity is comparable between male and female healthy volunteers. Proteasome chymotrypsin-like activity in isolated PBMCs was measured prior to any dosing of KZR-616 (N = 14 for females, 18 for males). Non-pre-dose-normalized enzymatic specific activity is presented as mean (± SEM). No significant differences were found between genders by Mann-Whitney test.

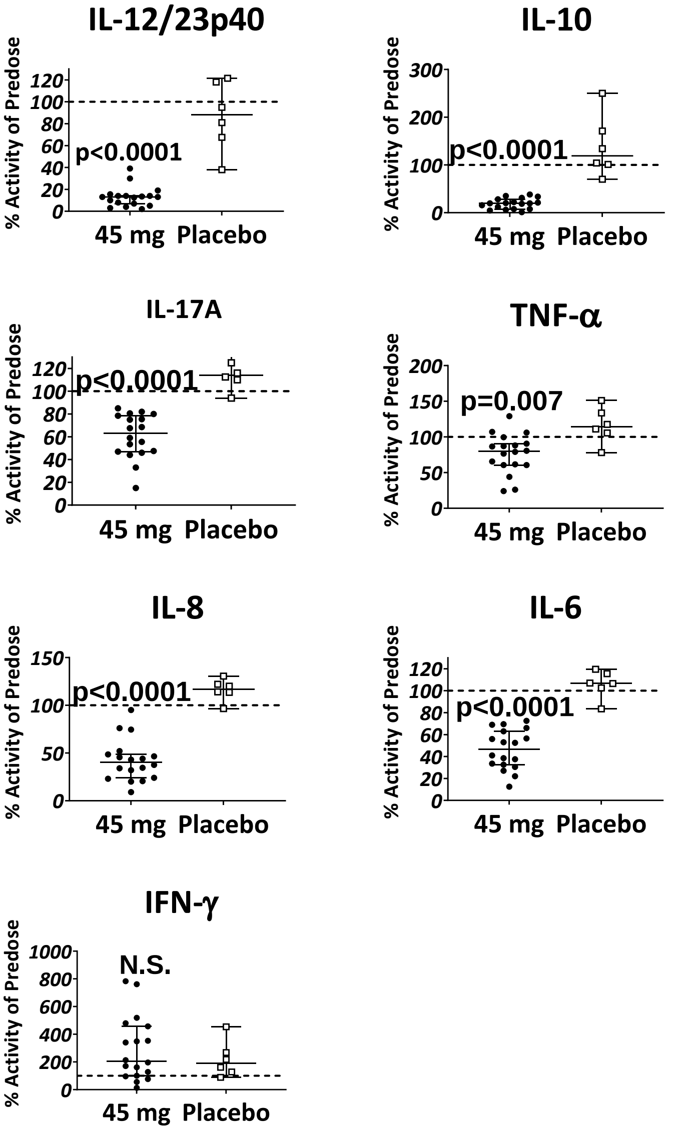

Supplemental Figure 11. KZR-616 administration to healthy volunteers blocks ex vivo stimulated cytokine release. Whole blood was drawn from subjects prior to and 4 h after administration of placebo or 45 mg KZR-616 and stimulated with PHA for 20 h ex vivo. Supernatants were analyzed for cytokines via multiplexed MSD immunoassay and normalized to pre-dose levels (dotted line). P values are by Mann-Whitney test.
