# Supplementary figures and images for: Zetomipzomib (KZR-616) attenuates lupus in mice via modulation of innate and adaptive immune responses
Source: Front Immunol. 2023 Mar 10;14:1043680. doi: 10.3389/fimmu.2023.1043680 (PMC10036830; doi:10.3389/fimmu.2023.1043680)

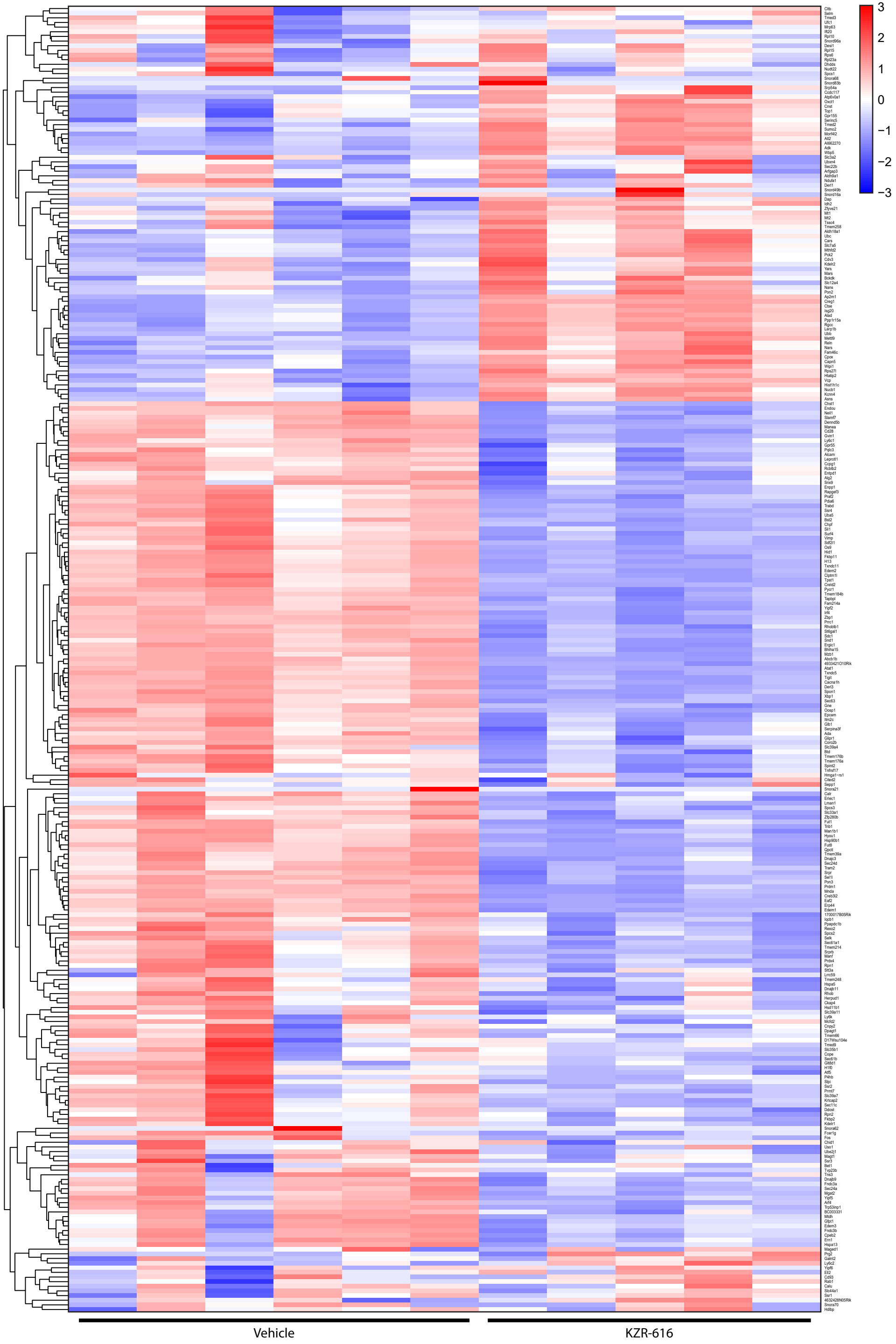

Supplement: Supplementary file 2 [file Image_8.pdf]
